# A Novel Computational Method Identifies Intra- and Inter-Species Recombination Events in *Staphylococcus aureus* and *Streptococcus pneumoniae*

**DOI:** 10.1371/journal.pcbi.1002668

**Published:** 2012-09-06

**Authors:** Lisa Sanguinetti, Simona Toti, Valerio Reguzzi, Fabio Bagnoli, Claudio Donati

**Affiliations:** 1 Novartis Vaccines and Diagnostics, Siena, Italy; 2 Dipartimento di Biologia Molecolare, Università degli Studi di Siena, Siena, Italy; 3 Istituto Nazionale di Statistica, Rome, Italy; The Centre for Research and Technology, Hellas, Greece

## Abstract

Advances in high-throughput DNA sequencing technologies have determined an explosion in the number of sequenced bacterial genomes. Comparative sequence analysis frequently reveals evidences of homologous recombination occurring with different mechanisms and rates in different species, but the large-scale use of computational methods to identify recombination events is hampered by their high computational costs. Here, we propose a new method to identify recombination events in large datasets of whole genome sequences. Using a filtering procedure of the gene conservation profiles of a test genome against a panel of strains, this algorithm identifies sets of contiguous genes acquired by homologous recombination. The locations of the recombination breakpoints are determined using a statistical test that is able to account for the differences in the natural rate of evolution between different genes. The algorithm was tested on a dataset of 75 genomes of *Staphylococcus aureus* and 50 genomes comprising different streptococcal species, and was able to detect intra-species recombination events in *S. aureus* and in *Streptococcus pneumoniae*. Furthermore, we found evidences of an inter-species exchange of genetic material between *S. pneumoniae* and *Streptococcus mitis*, a closely related commensal species that colonizes the same ecological niche. The method has been implemented in an R package, *Reco*, which is freely available from supplementary material, and provides a rapid screening tool to investigate recombination on a genome-wide scale from sequence data.

## Author Summary

The extent to which recombination occurs in natural populations is either unknown or controversial but it is widely accepted that recombination plays a crucial role in the evolution of many bacterial species. Numerous methods have been developed for the investigation of recombination events, but most of them require expensive computations and are applicable only to a limited number of genomes or to short nucleotide sequences. Here we present a new algorithm designed to identify recombination events affecting a group of adjacent genes. The procedure is based on the comparison of gene sequences and requires as input the matrix of gene conservation of a test genome against a group of reference genomes. The method is fast, and has minimal computational requirements. Therefore, it can be applied to datasets composed of a large number of complete genomes, and can be easily adapted to analyze data directly from high-throughput sequencing projects. We applied the algorithm to a dataset of *S. aureus* and streptococcal genomes and we found evidence of yet undetected inter and intra-species recombination events, suggesting that the use of *Reco* will shed new light on the evolution of bacterial species, and provide important information to improve classification criteria of bacterial species.

## Introduction

Recombination, the integration of foreign DNA in the chromosome of an acceptor cell, is one of the major evolutionary forces in bacterial species. Recombination can be mediated by viral infections [1], direct cell-to-cell contact [2] or transformation, when exogenous DNA is up-taken from the environment [3]. Homologous recombination occurs through the replacement of genomic segments with the homologous DNA from a donor of the same species, or from another, often closely related, species. Since the efficiency of RecA-mediated recombination decreases with increasing sequence divergence [4], recombination events are far more likely to occur between closely related DNA sequences, although homologous recombination is possible also when the sequence identity between the recipient and donor strains is as small as 70% [5]. In general, the incoming DNA must contain regions of high similarity to the recipient genome of length comprised between 25 and 200 bp to initiate DNA pairing and strand exchange [6].

Homologous recombination may involve whole genes or even larger segments. Whole genome sequencing has shown that homologous recombination is frequent in *Streptococcus pneumoniae* [7], [8], with a mean length of the insert of approximately 6.3 kb [9]. Exchanges of much larger DNA fragments occur in several other bacterial species. The distribution of Single Nucleotide Polymorphisms (SNPs) showed that isolates of *Streptococcus agalactiae* and *Clostridium difficile* can recombine DNA segments exceeding 300 kb with unrelated strains of the same species [10], [11]. Evidence of recombination involving the exchange of large chromosomal elements has been found in *Staphylococcus aureus*, involving the acquisition of sequences up to 557 kb [12]. These processes represent a major source of genomic diversity and are important in driving the emergence of clonal complexes and hypervirulent strains [10], [11], [12].

Numerous approaches have been developed to measure the frequency of recombination and to determine the chromosomal locations of the inserted sequences. Parametric methods estimate the recombination rate *ρ* = 4*N_e_r* (where *N_e_* is the effective population size and *r* is the per-base recombination rate) from the decline of linkage disequilibrium (the non-random association between alleles at different loci) with increasing distance along the chromosome [13], [14] by applying Markov Chain Monte Carlo methods [15] within the framework of coalescent theory [16], [17]. Hudson [18] developed a flexible, *ad hoc* method for estimating *ρ* by combining the coalescent likelihoods of each haplotype configuration for all pair-wise comparisons of segregating sites. Comparative methods that take into account the effects of recombination events on base composition, codon usage and base identity are the most common among non-parametric methods [19], [20]. Finally, phylogenetic methods infer recombination by comparing phylogenies from different parts of the genome, based on the assumption that high level of congruence among trees correlates with a lower frequency of recombination while little or no congruence is related to a higher rate of recombination [21], [22], [23]. Many of these algorithms for recombination detection have been combined in a single software package [24], [25]. These methods, although very accurate, are applicable only to relatively short and recently diverged sequences, for which an accurate multiple alignment is available.

Here, we introduce a novel method that, using a discrete filtering procedure of the gene conservation profiles in a panel of unrelated strains, identifies sets of contiguous genes likely acquired by homologous recombination. Due to its modest requirements in terms of computational resources, the method can be applied to large panels of complete genome sequences. The method was able to confirm known events of recombination involving genomes of *S. aureus*, accurately detecting the donor genomes and the genomic positions of the recombinant sequences. Additionally, the algorithm identified previously undetected recombination events in the genomes of *S. aureus* and *S. pneumoniae*. The filtering of sequence conservation data makes it feasible to also detect inter-species recombination events, where methods based on multiple sequence alignments are more difficult to apply. The method, implemented in the package *Reco*, provides a rapid and lightweight screening tool to investigate recombination on a genome-wide scale from sequence data.

## Materials and Methods

### Strain collection

*S. aureus*. A collection of 75 genomes of *S. aureus* (70 publicly available from NCBI and 5 newly sequenced) has been created including 71 strains isolated from humans and 4 strains isolated from cattle, sheep and swine. A complete list is reported in Table S1 and additional information on the newly sequenced genomes are reported in Table S2. *Streptococci*. A collection of 44 complete and draft genomes of *S. pneumoniae*, 4 genomes of *Streptococcus mitis*, 1 genome of *Streptococcus oralis* and 1 genome of *Streptococcus infantis* were downloaded from NCBI. A complete list of strains is reported in Table S3.

### Clonal complex designation

Clonal complexes were defined running eBurst [26] on the MLST databases for *S. aureus* and *S. pneumoniae* downloaded from the MLST website (http://www.mlst.net) in November 2010. In MLST, a Sequence Type (ST) is uniquely determined by the allelic profile at seven loci, *i.e.* internal fragments of the following genes *arcC*, *aroE*, *glpF*, *gmk*, *pta*, *tpi*, *yqiL* for *S. aureus*, and *aroE*, *gdh*, *gki*, *recP*, *spi*, *xpt*, and *ddl* for *S. pneumoniae*. Clonal Complexes (CCs) are groups of STs that share a recent common ancestor, defined by the eBURST algorithm by partitioning the MLST data set into groups of single-locus variants (SLVs), i.e., profiles that differ at 1 of the 7 MLST loci. This partitioning associates each ST with one CC and identifies the most likely founder ST, which is defined as being the ST with the greatest number of SLVs within the CC. Computed CCs were named after the ST of the predicted founder. For an example, see Fig. S1, where we report the structure of CC8 of *S. aureus*.

### Phylogenetic analysis

Phylogenetic analysis of the complete genome sequences has been performed using Mega4 [27]. The complete genome sequences have been aligned using Mauve [28]. From the region of the multiple genome alignments that are common to all sequences (the core genome) we have extracted the polymorphic sites, from which distance matrices were computed applying the Maximum Composite Likelihood Method implemented in Mega4. The trees were computed using the Neighbor Joining algorithm with 1000 bootstrap replicates.

### Gene comparison

The genes of each strain in the collection of 75 genomic sequences of *S. aureus* and of the 50 streptococci were aligned using FASTA version 3.5 [29] against all other strains in the same collection. Orthologs were identified using the reciprocal-best-hit algorithm with lower bounds of 75% identity on 75% of the sequence length. In general, other choices are possible. The only requirement of the method is that there is a 1-to-1 correspondence between the genes in each genomic sequences. In the case of nearly identical reciprocal-best-hits the regions harboring these should be treated separately. Being based on comparison between genes, the algorithm does not distinguish if a gene is located in different regions in the different strains. This feature allows to study recombination events also in cases where a complex series of rearrangements occurred after the recombination event, and to use directly data from Next Generation sequencers, where the assembly of long contigs can be problematic. However, where possible, the user should directly check the position of the genes involved in the putative recombination on the genomic sequences. Using these data, for each genome we computed the identity matrix 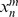 , where 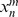 is the pair-wise percentage of identity of the *n-*th gene with the homologous gene in the *m-*th genome.

### Recombination detection algorithm

The purpose of the algorithm is the identification of recombination events affecting groups of adjacent genes in a genomic sequence. In the following, we will define as “recombinant” the strain(s) containing the recombinant segment, as “major parent” the strain(s) contributing the genetic backbone of the recombinant strain, and as “minor parent” the strain(s) contributing the sequence that was inserted into the backbone by the recombination event. The identification of recombination events affecting more than one gene has to face two obstacles, which are related to the age of the event itself, *i.e.* the amount of time since the event occurred, and the subsequent evolution of the sequence. Neighboring genes, coding for proteins with different functions and experiencing widely different selective pressures, can evolve at different rates, progressively erasing the phylogenetic signals of recombination. Moreover, later recombination events can superimpose on older ones, resulting in fragmented patterns, which are difficult to reconstruct. To overcome these difficulties, we designed a digital filter that first performs a smoothing of the conservation data of neighboring genes, and then reduces the smoothed data to a binary scale. Using this filter, the fragmented pattern is averaged out and the signals from the events larger then a given length scale are enhanced. Since this scale is determined by a free parameter of the algorithm (the sliding window size *l*, see below), using different values of the parameters can lead to a clearer picture of the signal present in the data. The discretization is based on the gene-specific distribution of sequence conservation, and is therefore independent from the rate of evolution of single genes. The procedure can be schematically divided into the following steps:

#### Step 1: Detection of putative donor genomes

The purpose of this step was to identify sets of contiguous genes with an anomalous level of conservation compared to one or more reference genomes, independently from the rates of evolution of the individual genes.

Initially, we selected one test genome, for which we wanted to identify the genes recently acquired by homologous recombination. For this genome, we computed the matrix 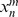 of the sequence conservation of the *n*-th gene in the *m*-th reference genome ( 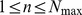 where 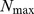 is the total number of genes in the *m*-th reference genome). To eliminate short-range fluctuations in the conservation profile and restrict our search only to recombination events exceeding a given size, for circular genomes we defined 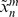 as:(1)

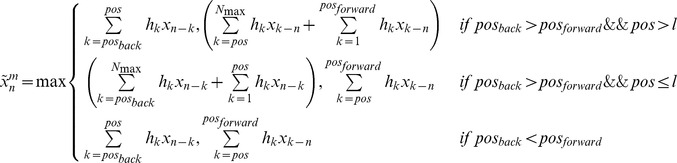

where:

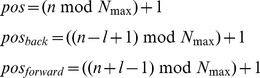

and where 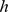 is a weighting function, *l* is the length of the sliding window over which the short range fluctuations are averaged, and mod indicates the modulus function, *i.e.* a mod b is the remainder of the integer division a/b.

For linear genomes 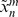 is defined as:

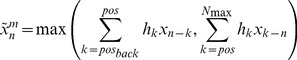

(2)
In the language of signal theory the sliding window weighted average in (1) or (2) is equivalent to implementing a digital filter, and the effect of the filtering depends on the choice of the size of the sliding window *l* and of the weighting function 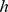 [30]. Although any function with a compact support could be used as a weighting function, in the following we have used a modified *rect* function:

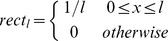

where the 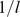 factor guarantees the normalization, so that the value of 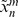 is the average of 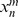 over a sliding window of size *l*, a procedure commonly used in signal preprocessing (see, for instance, [31]). When a gene is absent, the average is computed only over the present genes, and the normalization factor is modified accordingly. The average 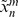 is then used for the whole region. While careless use of this procedure can lead to incorrect conclusions, direct inspection of the putative recombinant region should allow the user to easily identify the absent genes. On the other hand, this procedure allows the identification of recombining regions also in the presence of insertions or deletions that occurred after the recombination event and that could obscure the recombination pattern. This moving average approach is a compromise between the need to reduce the noise affecting the individual points, and the attempt to achieve maximum sensitivity to localized variations in the data.

Beside the *rect_l_* function, other weighting functions (*Gaussian* and *sinc* function) have been tested. The results obtained with these alternative choices were comparable, and these functions can be chosen as alternative filtering methods in the software implementation of *Reco*. The size of the support of 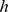 was tuned according to the size of the recombination events that we wanted to identify, and, except where otherwise stated, was set equal to 25 genes. In general, given that 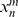 is averaged over a sliding window of size *l*, events involving less than *l* genes are averaged out by this step. Choosing the right value of *l* is essential for the efficacy of the method, and several choices should be tested by the user.

To visually identify regions of the target genome showing an anomalous pattern of sequence conservation with the reference genomes, we then converted the filtered matrix 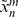 into a discrete 0–1 matrix by the following rule:

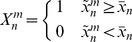

where 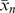 is a gene-specific cut-off value. In general, other strategies to highlight the regions where there is a change in the pattern of sequence relatedness between the sequences of the same size are possible. One possibility would be ranking the sequences according to their similarity to the test strains. However, in the case of highly conserved sequences, this method is very sensitive to random sequence variation due to genetic drift, and could yield a high rate of false positives. The optimal value of 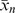 is indirectly influenced by the number and genetic relatedness of the genomes included in the analysis. In the following, 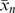 is set to the value of the limit of the third quartile of the distribution of 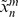 over the 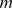 genomes, but a lower cut-off value 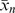 should be set to correct for the sampling bias when the dataset includes closely related sequences.

The choice to discretize the data using the gene-dependent distribution of sequence conservation allowed us to correct for differences in the natural rate of evolution between different genes. In this way, while the actual value of the cut-off for sequence conservation was different for each gene and depended on the gene-specific mutation rate, regions of the test genome having an anomalously high level of conservation with the reference sequence were readily identified.

#### Step 2: Breakpoints detection

Breakpoints of putative recombination events could be visually identified in the matrix 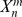 since they corresponded to points where the profile of conservation of the test genome compared with the reference sequences changed sharply. To automatically detect the position of the breakpoints for each position 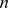 along the test genome and each reference genome 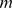 , we computed the proportions 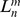 and 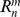 of values 1 in the intervals 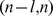 and 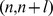 in the matrix 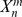 , respectively. The null hypothesis 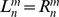 was tested using a Fisher exact test (fisher.test function in R http://www.r-project.org/). To minimize the number of parameters, *l* was set of the same size as the sliding window used for the filtering step, although *Reco* allows the user to choose other values. Considering that the test was applied to a discrete 0–1 matrix, minima in the p-values lower than 0.05 could be considered reliable recombination breakpoints.

#### Step 3: Selection of the optimal sliding window size

**Figure 1:**
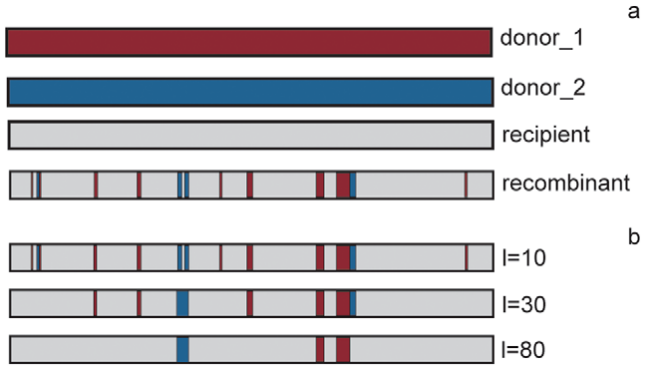
Schema of the artificial recombinant genome. a) The recombinant genome has been designed identical to a major parent (recipient) and containing segments acquired from donor_1 (red) and donor_2 (blue) in a variable number. b) Recombination events detected for different sizes of the sliding window (top to bottom, *l* = 10, *l* = 30, *l* = 80).

While many efficient methods exist to identify small recombination events in a set of short aligned sequences, few convenient methods exist to identify on a genome-wide scale large events, including several genes or entire operons. The proposed procedure, using a smoothing of the conservation signal over a window of size *l*, is especially designed to identify large events, including at least *l* genes, and therefore the user should carefully evaluate different values of the parameter. To qualitatively characterize the influence of the size *l* of the sliding window on the sensitivity of the algorithm, we created an artificial genome in which we introduced thirteen recombination events of known lengths and positions. Specifically, the recombinant genome was identical to a major parent (*S. aureus* NCTC 8325) except for thirteen recombination regions of length comprised between 10 and 100 genes (Fig. 1), that were substituted with the homologous sequences from two distinct donor genomes, donor_1 and donor_2 (*S. aureus* MRSA252 and *S. aureus* MSSA476). We analyzed the matrix 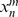 of the recombinant genome against 5 reference genomes, *i.e.* the major parent, donor_1, donor_2 and two test genomes (*S. aureus* JH9 and *S. aureus* RF122), with different value of 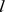 . The full panel of recombination events was visible for 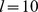 . However, for this value of 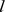 there were few highly conserved areas in the genome of the recipient strain that, due to small fluctuations in the level of sequence conservation, were misclassified as recombinant (data not shown). Increasing 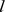 these false positives were eliminated, but also short recombination events remained undetected. By setting 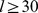 , the algorithm detected all the recombination events greater than 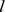 , effectively behaving as a low pass filter [30]. Also, for 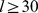 , two distinct recombinant regions of 40 genes at a distance of 10 genes acquired from donor_2 were identified as a single recombination event including 90 genes.

*Reco* has been implemented as an add-on package for the statistical software R (http://www.r-project.org/) available as Supplementary Protocol S1 (see also the Help provided as Supplementary Text S1).

## Results

### Recombination in *S. aureus*

*S. aureus* is a major cause of nosocomial infections, including bacteremia, metastatic abscesses, septic arthritis, endocarditis, osteomyelitis, and wound infections. However, its pathogenic mechanisms have not yet been fully elucidated. In particular, the factors that render certain strains, such as the methicillin resistant (MRSA) strain TW20, highly transmissible and invasive have not yet been identified [12]. Horizontally acquired DNA could represent a critical element of the evolution and acquisition of virulence mechanisms in *S. aureus*. Although the estimated rate of recombination is lower than in other pathogenic bacteria like *Neisseria meningitidis* and *S. pneumoniae* [22], events of recombination involving unusually large portions of the genome have been identified [32]. To test the effectiveness of our method, we have analyzed a collection of 75 genomes of *S. aureus* (70 available in the public domain and 5 sequenced for this study, Table S1), identifying several recombination events, some of which had not been reported before.

#### *Reco* identified one large chromosomal recombination event in *S. aureus* ST239 strains

**Figure 2:**
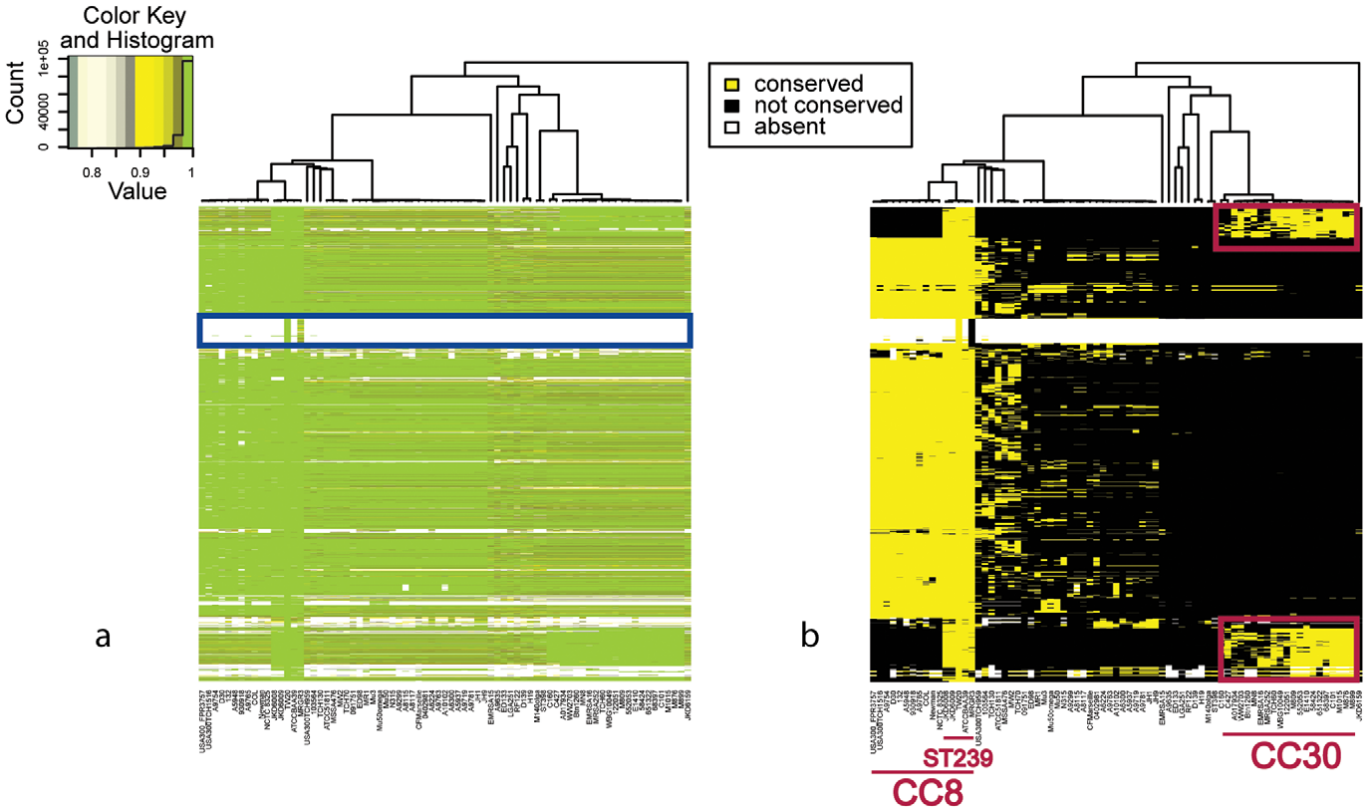
Recombination in *S. aureus* ST239. a) Heatmap representation of the percentage of conservation of the genes of *S. aureus* TW20 strain (ST239) against all the other strains. Each row represents the percentage of identity of one gene in TW20 in all other strains. Each column represents one reference genome. The origin of replication of TW20 is at the bottom of the figure, and rows are ordered according to the gene order in TW20. Columns are ordered according to a whole-genome phylogenetic tree (upper panel). The color code and a histogram of the percentage of identity of the TW20 genes in the reference genomes are shown in the inset. The blue box identifies a phage shared by TW20 and MRGR3 and not present in other strains. b) Filtered data. Yellow and black dots indicate conserved and non-conserved genes, respectively. White dots indicate missing genes. Red boxes highlight two regions that TW20 acquired from one CC30. Due to the circular nature of the chromosome of *S. aureus*, these two regions have probably been acquired in a single recombination event.

**Figure 3:**
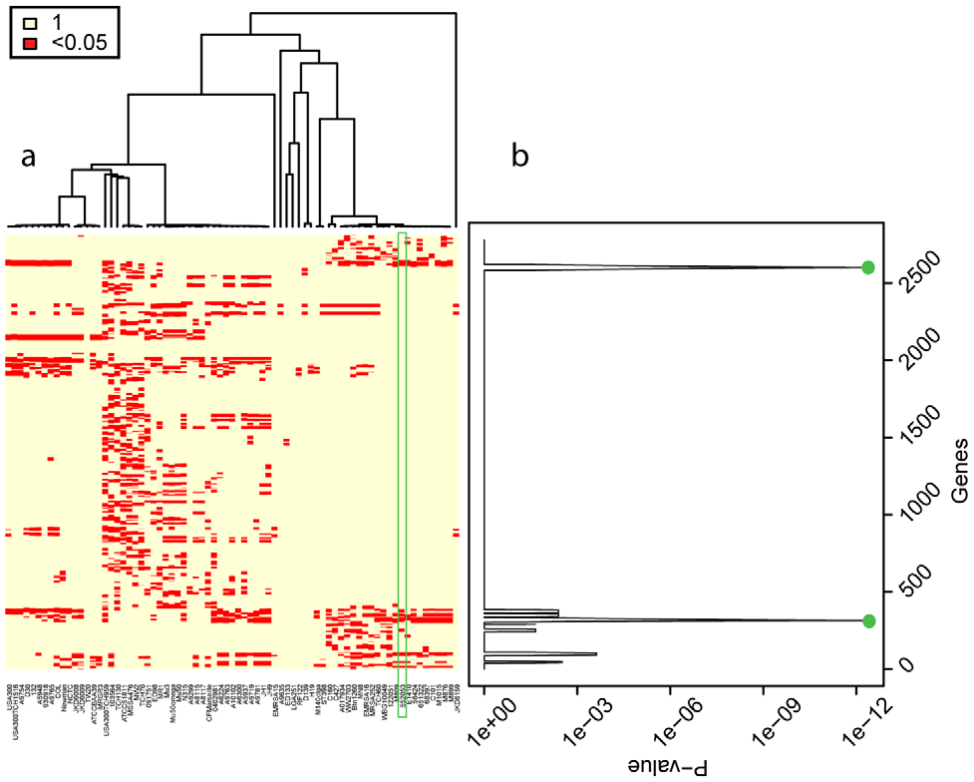
Borders of the recombinant region in ST239. a) P-values of the Fisher test performed on the filtered matrix obtained from the comparison of TW20 (ST239) against all the other *S. aureus* strains. b) P-values of the Fisher test of TW20 against 552053 (CC30). Minima in the p-values (green dots) identified the breakpoint positions of the recombination event shown in Fig. 2b.

Strains of Sequence Type ST239 (classified by eBURST into CC8, see Fig. S1) display evidence of a large recombination event involving a region of approximately 557 kb spanning the origin of replication, that was likely acquired from a CC30 strain [32]. In order to test the ability of our procedure to identify this event, we analyzed the TW20 (ST239) strain. The degree of conservation of the genes of TW20 compared to all the other genomes of *S. aureus* in our collection is high (Fig. 2a). On the basis of these data we could identify the presence of a region encoding for a phage not shared with other strains. Identifying this feature does not require processing of the data, as it is already visible from the presence/absence profile by using the visualization tool provided by *Reco* (blue box in Fig. 2a). However, other recombination events distinguishing TW20 from the other CC8 strains were obscured by noise. In Fig. 2b we show the data after the filtering step. While, as expected, most genes of ST239 had their closest homologs in CC8 strains (Table S1), we identified two regions located on opposite sides of the origin of replication (red boxes in Fig. 2b) that were closely related to the homologous regions in CC30 strains (Table S1). These two regions are contiguous since the chromosome is circular, and thus are likely to have been acquired in a single event. To determine the borders of the recombinant region (or recombination breakpoint positions), we computed the matrix of the p-values of the Fisher test (see Methods) for the TW20 (ST239) strain against all the other strains (Fig. 3a). Among the p-values, the minima (see Table S4) identified the exact breakpoint positions as shown in Fig. 3b, where we report the p-values against the 552053 (CC30) strain only. The data support the presence of a large recombination event around the origin of replication on both sides of the minima (highlighted in green). Peaks in the p-values near the origin are due to the loss of some genes in strain 552053.

#### Identification of horizontal gene transfer events within the *S. aureus* Clonal Complex 10

The strain H19 (ST10, CC10) showed two regions, consisting of about 50 ORFs and encoding for two phages, not conserved in the closely related strain D139 (ST 145, CC10) (Fig. S2). To visually identify the donor strains for the recombination events, in Fig. S3a we reproduced the image with a subset of potential donor strains and we found that the two regions were exchanged in two distinct recombination events: strains of CC30 or CC1 were the minor parent in one case (region 1 in Fig. S3a), while strains of CC5 were the minor parent in the other (region 2 in Fig. S3a). Interestingly, CC30 and CC1 strains contain two more phages, that are closely related to those contained in the homologous regions of strain D139 (region 3 and 4 in Fig. S3b, that are homologous to region 1 and 2 in Fig. S3a, respectively). Therefore, strain D139 appears to have acquired two phages from CC30 or CC1 and from CC30, respectively, that are not related to the ones present in H19 (Fig. S3b), but nevertheless are inserted in regions where strain H19 also harbors unrelated phages, suggesting that these regions represent hotspots for phage insertion, and highlighting the complex dynamics of phage exchange in *S. aureus*.

#### Identification of a recombining strain within Clonal Complex 5

**Figure 4:**
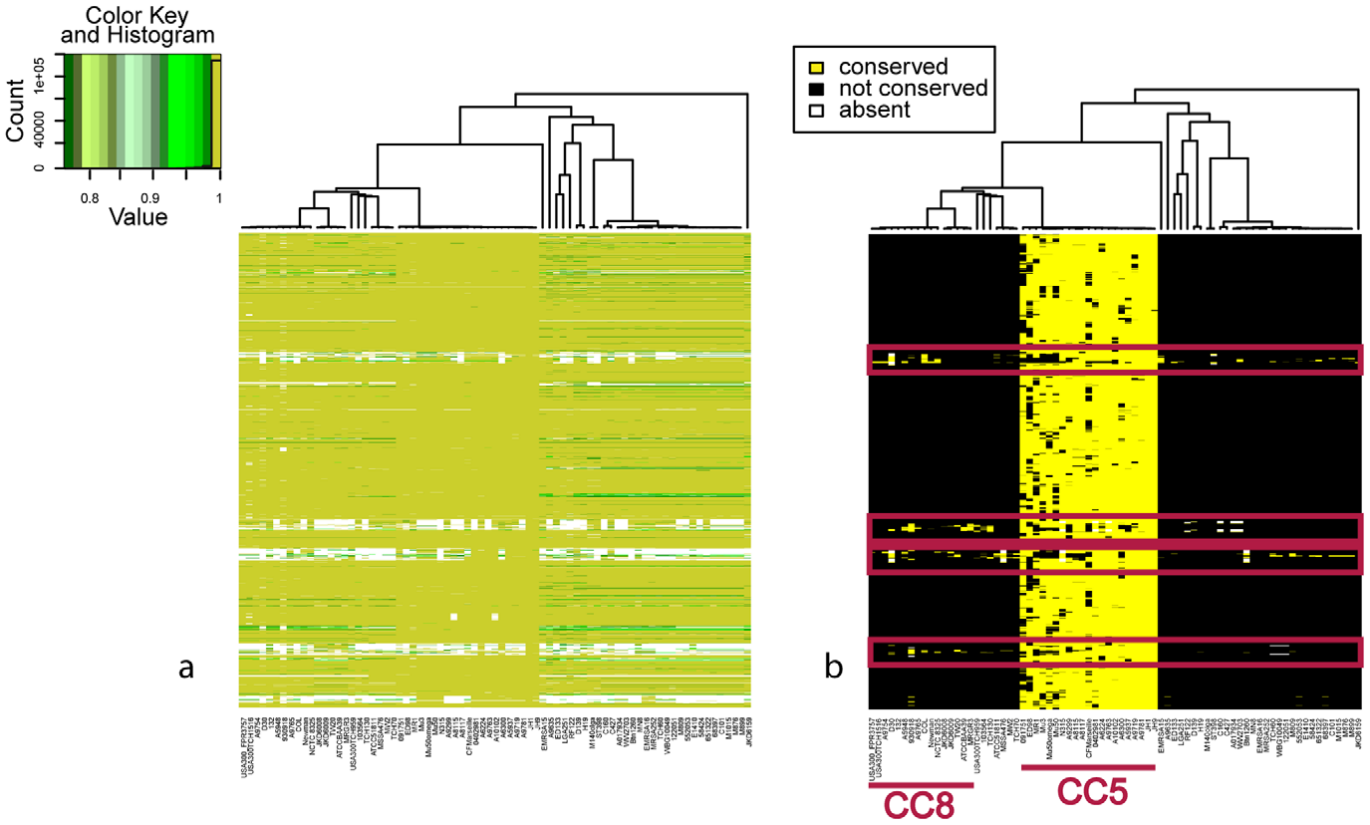
Recombination in *S. aureus* CC5. a) Comparison of *S. aureus* JH1 (ST5, CC5) strain with all the other *S. aureus* genomes. b) Filtered data. Red boxes represent the four regions not conserved in most of CC5 genomes, corresponding to phage encoding islands acquired by a JH1 ancestor after the diversification from the other CC5 strains.

CC5 is one of the most prevalent Methicillin-resistant (MRSA) lineages of *S. aureus* worldwide [33]. Unlike other CCs, *S. aureus* genomes of CC5 in our collection were highly conserved, probably due to a sampling bias. Despite this fact, we identified four potential cases of horizontal gene transfer in the ST5 strain JH1 that differentiate these strains from other strains of the same CC (Fig. 4). The regions correspond to the four phages harbored by JH1, consisting of about 50 genes each. The conservation pattern in Fig. 4 showed that the phages of JH1 are closely related to phages found in the genomes of the CC8 strains, although it is evident from the data that a complex pattern of recombination has occurred.

### Recombination in *S. pneumoniae* and related species

*S. pneumoniae* is the causative agent of several human diseases, which include chronic otitis media, sinusitis, pneumonia, septicemia, and meningitis. *S. pneumoniae* is a naturally competent organism that is known to easily transfer genetic material both within the species and from closely related species. Despite evidences of extensive recombination, it has recently been shown that in a phylogenetic analysis using whole genome sequences, strains of the same ST or CC always form a monophyletic branch [7], suggesting that recombination events are not able to destroy the phylogenetic signal contained in whole genome sequences. We analyzed a dataset of 50 genomes including *S. pneumoniae*, *S. mitis*, *S. oralis* and *S. infantis*, to identify putative recombination events.

#### Horizontal gene transfer within the *S. pneumoniae* Clonal Complex 306

**Figure 5:**
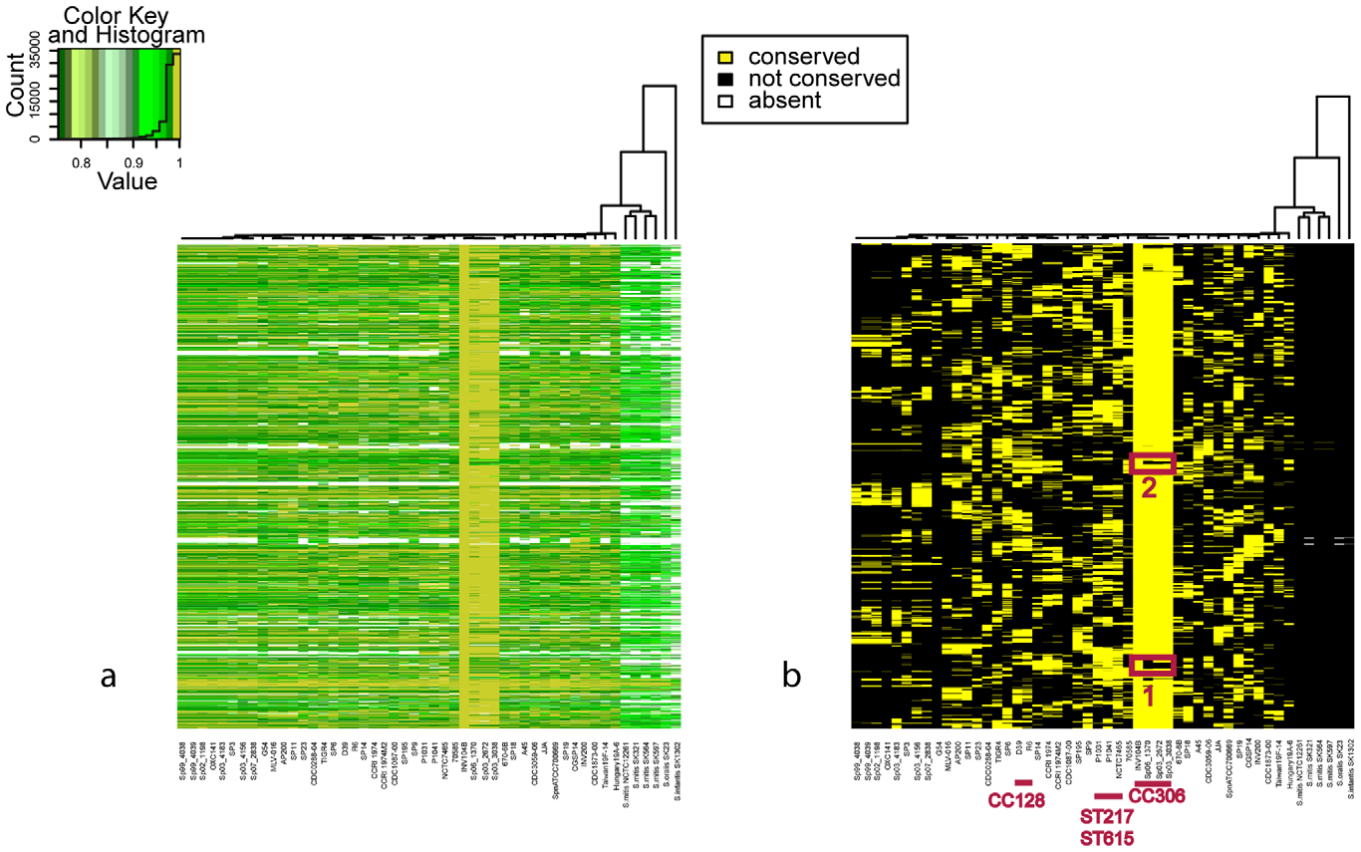
Recombination in *S. pneumoniae* CC306. a) Comparison of *S. pneumoniae* INV104B (CC306, Serotype 1) with all the other *S. pneumoniae* strains. b) Filtered data. INV104B shows two regions not conserved in other CC306 (red boxes 1 and 2). Region (1) show similarity with the genomes of ST217 (P1031, P1041) and ST615 (NCTC7465) strains, while region (2) is conserved in CC128 strains (D39, R6).

We found two regions of the *S. pneumoniae* INV104B strain (ST227, CC306) that were not conserved in other CC306 strains (Fig. 5 boxes 1 and 2). The region 1 includes about 33 genes that INV104B acquired from either ST217 or ST615 strains, or from a common ancestor of the two. The recombinant region is located upstream of the capsule biosynthesis (cps) locus and includes genes involved in polysaccharide synthesis like, for instance, the SPNINV104_02650 gene, coding for the sugar phosphate isomerase, that is required for capsule formation [34]. Interestingly, both the recombinant and the putative minor and major parents are of capsular Serotype 1. Due to the high level of conservation of the cps locus between these strains, it was not possible to ascertain whether the latter was transferred in the same event. The second potential recombination event (Fig. 5 red box 2) consists in about 12 genes that INV104B acquired from D39 or R6, two closely related strains belonging to CC128.

**Figure 6:**
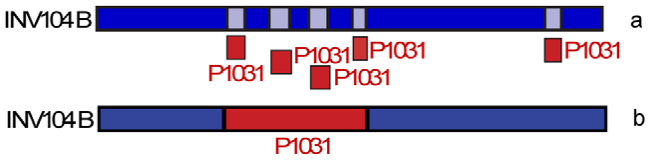
Recombination events in *S. pneumoniae* strain INV104B identified using RDP. a) RDP detected four regions acquired from strain P1031. Strain P1031 belongs to CC217 and it could be the putative donor strain for INV104B. b) *Reco* grouped the recombination events of INV104B in a single event (Fig. 5b region (1)).

In order to highlight the specific features of *Reco*, we applied RDP3 [24], [25] to the region upstream the capsule biosynthesis locus of the *S. pneumoniae* INV104B strain (CC306), where *Reco* identified a putative recombination event with the P1031 strain as donor strain. In this region RDP3 found four segments of recombination potentially acquired from P1031 (Fig. 6 a), while *Reco* identified a single event spanning the entire region (Fig. 6 b). The different results between RDP3 and *Reco* shown in Fig. 6a,b highlight some of the specific features of the smoothing procedure employed by *Reco*. While RDP3 accurately identifies the borders of the regions containing the signal for homologous recombination, *Reco* extends the signal also to neighboring regions, therefore “averaging out” discordant signals over regions including less than *l* genes (*l* is the sliding window size fixed by the user). This feature, that in some cases can lead to incorrect results, allows *Reco* to recognize events that are artificially split into many smaller events by other algorithms due either to subsequent, smaller recombination events that have superimposed on the original one, or to the presence of highly conserved regions, as in the cases of genes under purifying selection, where there is no signal for recombination. Direct inspection of the multiple alignment of the whole segment considered in Fig. 6a,b shows that the regions separating the four recombination events detected by RDP3 are perfectly conserved between the putative major parent, minor parent and recombinant strains. While RDP3 accurately detects the border of the regions where there is direct evidence of recombination, we cannot rule out that also the conserved regions where transferred. In fact, the latter possibility is the most likely assuming parsimony. However, in general only the careful comparison of more than one method can allow to draw definitive conclusions. *Reco* should be considered as a rapid screening tool to identify regions that should later be studied using other, more sensitive tools.

#### Horizontal gene transfer within the *S. pneumoniae* Clonal Complex 15

We found three regions of the ST15, CC15 strain CGSP14 that were not conserved in INV200, the other CC15 strain present in the collection. The first region (Fig. S4 red box 1) consists of 69 genes encoding for a large conjugative transposon of 68 kb [28] inserted near the *rplL* gene. This region is absent from most of the strains in our panel, and is probably imported from a strain closely related to SpnATCC700669 (CC81, serotype 23F) that harbors an highly conserved homologous region in the same genomic position [9]. The second region (Fig. S4 red box 2) not conserved in INV200 consists of 20 genes containing the *psrP*-*secY2A2* pathogenicity island, that was found to correlate with the ability of *S. pneumoniae* to cause invasive disease [35]. PsrP is a large ∼4500 amino acid serine-rich protein not present in all pneumococci and located near glycosyltransferases that are probably involved in its modification. The recombination event identified included a contiguous block of genes probably acquired from a strain closely related to CDC3059-06 (CC199) and TIGR4 (CC205). The third block of genes (Fig. S4 red box 3) was found conserved in SpnATCC700669 and in most of the genomes of CC180. This region included *pcpA*, coding for a choline binding protein suggested to be an adhesin [36] and known to be important for virulence. The position of the putative recombination breakpoint positions in *S. pneumoniae* CGSP14 and the putative donor strains are reported in table S5.

#### Inter-species recombination in the *S. pneumoniae*-*S. mitis* complex

*S. pneumoniae* and closely related streptococcal species are known to colonize the same habitat and to actively exchange genetic material, often including genes implicated in antibiotic resistance [37]. Whole genome comparative analysis has shown that as much as 30% of the sequence variability in the part of the genome of *S. pneumoniae* shared with *S. mitis* could be due to homologous recombination with the latter [7]. To identify occurrences of inter-species exchange of genetic material between pneumococci and commensal streptococci, we compared the available genomes of *S. pneumoniae* with the genomes of three closely related species (four strains of *S. mitis*, one strain of *S. oralis*, and one strain of *S. infantis*).

**Figure 7:**
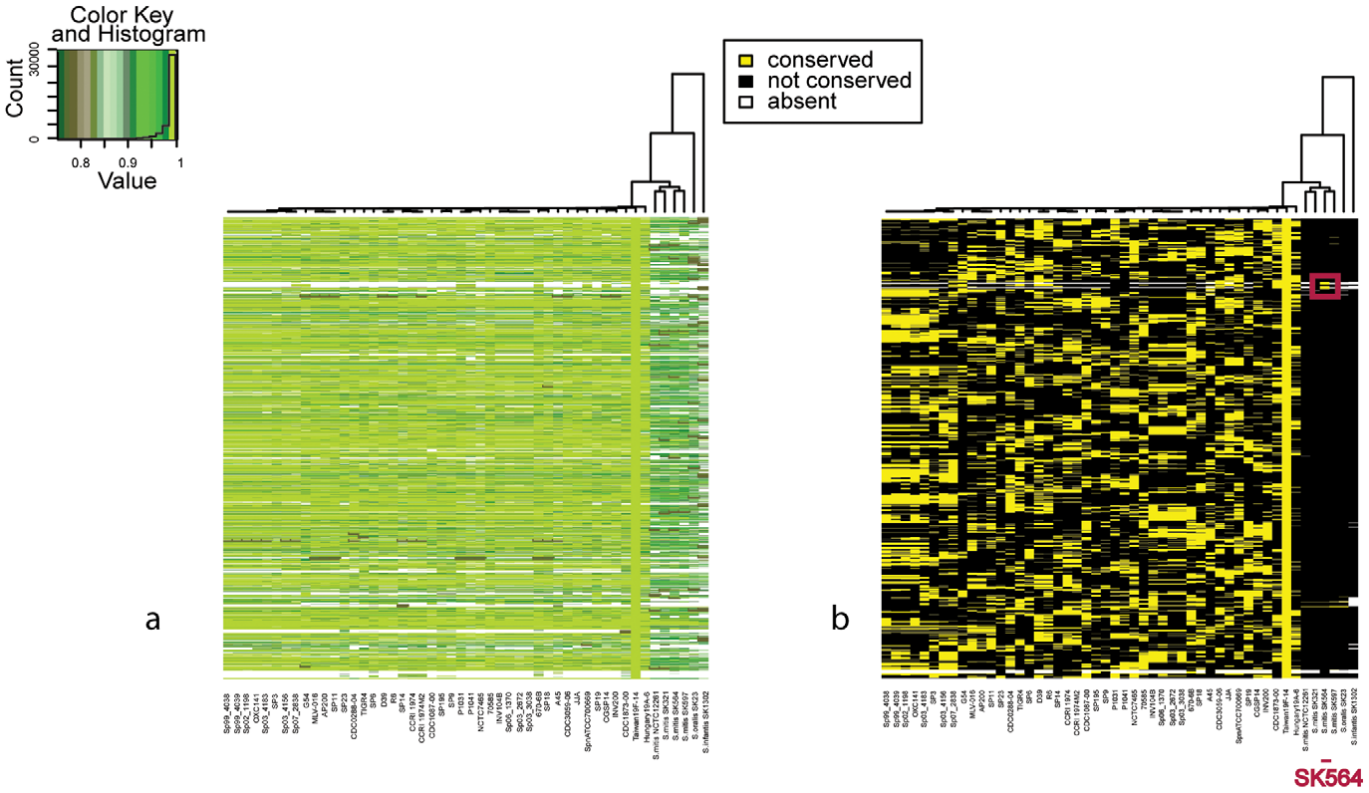
Recombination in the *S. pneumoniae*-*S. mitis* complex. a) Comparison of *S. pneumoniae* Taiwan19F-14 (ST236, CC271) strain against all the other streptococci. b) Filtered data. Red box represents a region of Taiwan19F-14 shared with *S. mitis* SK564 with a percentage of conservation higher than many strains of *S. pneumoniae*. This region includes a transposon coding for erythromycin resistance and represents an event of horizontal transfer between two closely related species both colonizing the nasopharynx of human hosts.

We found one case of exchange between the antibiotic resistant *S. pneumoniae* Taiwan19F-14 strain (ST236, CC271) [38] and the *S. mitis* SK564 strain (red box Fig. 7). The region consists of ∼66 kb including 23 ORFs carried on a transposon (see Table S6). While most of the ORFs included in the transposon were absent in all *S. mitis* strains, a portion of the transposon, for a total of 4 kb encoding for erythromycin resistance is highly conserved between *S. pneumoniae* Taiwan19F-14 and *S. mitis* SK564. Interestingly, the gene SPT_1926 annotated as macrolide efflux protein, as well as several of the genes both upstream and downstream of the transposon were highly conserved, but were found to be frameshifted in the genome of SK564 (see Table S6), suggesting that this sequence was transferred from *S. pneumoniae* to *S. mitis*, where the genes not conferring a selective advantage have lost their coding ability due to point mutations [34]. Some of the genes located upstream and downstream of the transposon in Taiwan19F-14 that were highly conserved in SK564, were found to be not-contiguous, and located in other regions of the SK564 genome, suggesting a complex evolutionary history of this region after homologous recombination from a strain closely related to the antibiotic resistant *S. pneumoniae* Taiwan19F-14. A possible explanation could be that the transfer occurred in one step from *S. pneumoniae* to *S. mitis*, followed by a translocation event mediated by the transposon. Most of the genes on the transposon were subsequently lost, while some were inactivated and will probably be lost in the future. This event is just an example of the widespread exchange of genetic material between *S. pneumoniae* and *S. mitis*, that has been suggested to constitute the main reservoir of genetic variability for the pneumococcus [7], [39].

## Discussion

Population genetic studies on many bacterial species, such as *N. meningitidis*, *S. aureus* and *S. pneumoniae*, have provided extensive evidence of the exchange of genetic material amongst unrelated strains. Knowledge of basic parameters, including the population size, as well as the mutation, recombination and migration rates might help us to predict the extent to which genes are exchanged amongst strains within the same population and between geographically separated populations of a species. In particular, for pathogenic microorganisms this information might help us to understand the dynamics of drug resistance spread, the evolution of vaccine escape mutants, and, more generally, the evolution of pathogenicity. Several methods for the identification of recombination events in sequence data are present in the literature. However, these methods are computationally demanding and difficult to use on whole genome sequences. On the other hand, the rapid diffusion of Next Generation Sequencing technologies requires the development of lightweight, user-friendly algorithms and tools for sequence analysis. Here we presented a new method to identify recombination events in genomic sequences that can be readily applied to large sequence datasets.

We have applied the algorithm to two pathogens, *S. pneumoniae* and *S. aureus* that are reported to have widely different propensity to exchange genetic material [7], [40]. Although many studies have indicated that *S. aureus* has a low recombination rate [41], we found evidences of several recombination events, also involving large portions of the genome. Except for the large chromosomal replacement found in ST239, most of these events involved highly mobile elements, particularly phage encoding islands. Differently, most of the recombinant regions identified in *S. pneumoniae* and its related species contained surface proteins or virulence factors suggesting that these regions are exchanged more frequently than others, possibly due to the selective pressure from the host immune system and confirming that genes involved in DNA replication, transcription and translation are less likely to be horizontally transferred [42]. We found one recombination event potentially including the capsular biosynthesis locus of Serotype 1 strains. Interestingly, both the donor and acceptor strains are of Serotype 1. It had already been noted that, differently from other serotypes, the Serotype 1 capsule is found only in a group of clonally related strains [7]. This finding could be due to a general inability of Serotype 1 strains to participate to the exchange of genetic material, either as donor or as acceptor. The event identified here rather suggests the existence of a unknown barrier to recombination with strains of unrelated Serotypes, possibly due to the peculiar lifestyle of Serotype 1 strains, that are very rarely carried and are commonly retrieved only from invasive disease [43].

Extending the analysis to the *S. pneumoniae*-*S. mitis* complex we identified an example of interspecies recombination that involved contiguous genes that were transferred and maintained in the host genomes. While all genes involved in this event are functional in *S. pneumoniae*, many of them were pseudogenes in *S. mitis*, suggesting that this region was recently imported into *S. mitis* from *S. pneumoniae* and that genes that do not confer a selective advantage to the former are in the process of being purged. It has been estimated that as much as 30% of the genetic diversity of *S. pneumoniae* could be attributed to homologous recombination with *S. mitis* [7], and the extent of genetic exchange within the *S. mitis*-*S. pneumoniae* complex has lead to the hypothesis that *S. mitis* is the genetic reservoir of *S. pneumoniae* [39].

The rate of homologous recombination varies greatly between different species [44], and between different lineages of the same species [45]. The effects of homologous recombination on the evolution of bacterial species are profound, and far from being fully understood. The advent of Next Generation Sequencing technologies is providing an unprecedented amount of sequence data that, due to their complexity, cannot be analyzed using available methods. We introduced a simple algorithm that is able to handle large amounts of data and could provide a rapid screening tool to globally investigate recombination from sequence data.

## Supporting Information

Figure S1 **Structure of CC8 of** ***S. aureus*** **.** Minimum spanning tree of the CC8 obtained by eBurst on the MLST data. The STs included in our strain collection are indicated.
(PDF)

Figure S2 **Recombination in** ***S. aureus*** **CC10.** a) Comparison of *S. aureus* H19 (CC10) strain against all the other *S. aureus* strains. b) Filtered data. Two regions (box 1 and 2) of H19 are not conserved in the other genome of CC10 (D139). These regions include two phages shared with CC10 strains.
(TIF)

Figure S3 **Comparison of H19 with the subset of potential donor genomes of regions 1 and 2 in Fig. S2b.** a) Region (1) shows high similarity with genomes that belong to CC30 (122051, MRSA252, WBG10049) or CC1 (ATCC51811, MSSA476, MW2) while region (2) shows high similarity with genomes of CC5 (Mu3, Mu50omega, Mu50, N315, 0402981, A9763, A10102). b) Comparison of D139 with the subset of potential donor genomes. Region (3) was probably acquired from genomes of CC30 (122051,MRSA252, WBG10049) or CC1 (ATCC51811, MSSA476, MW2), while region (4) was probably acquired from genomes of CC30 (122051, MRSA252, WBG10049).
(TIF)

Figure S4 **Recombination in** ***S. pneumoniae*** **CC15.** a) Comparison of *S. pneumoniae* CGSP14 (CC15) strain against all the other *S. pneumoniae* strains. b) Output of the low pass filter. CGSP14 shows mainly three large regions not shared with the other strain INV200 belonging to CC15. Region (1) shows similarity with SpnATCC700669 (CC81, serotype 23F), region (2) with CDC3059-06 (CC199) and TIGR4 (CC205) and region (3) with SpnATCC700669 and most of the genomes of CC180.
(TIF)

Protocol S1 ***Reco*** **package.** The *Reco* package implemented as an add-on package for the statistical software R (http://www.r-project.org/).
(GZ)

Table S1 **Strain collection of** ***Staphylococcus aureus***.
(DOC)

Table S2 **Newly sequenced strains of** ***Staphylococcus aureus***.
(DOC)

Table S3 **Strain collection of** ***Streptococcus pneumoniae***.
(DOCX)

Table S4 **Recombination breakpoint positions of TW20.** The first column reports the gene name of the potential recombination position in the test genome TW20, the second column the reference genome and the third column the corresponding p-value of the Fisher test.
(XLS)

Table S5 **Recombination breakpoint positions of CGSP14.** First column reports the gene name of the potential recombination position in the test genome CGSP14, the second column the reference genome and the third column the corresponding p-value of the Fisher test.
(XLS)

Table S6 **Inter species recombination.** We report the id, functional annotation and conservation of the genes involved in an inter-species recombination event involving the antibiotic resistant *S. pneumoniae* Taiwan19F-14 strain and the *S. mitis* SK564 strain. In yellow we report the genes coded by the transposon, while in red we highlight the genes that are frameshifted in *S. mitis*.
(XLS)

Text S1 **Help file for the** ***Reco*** **package.**
(DOC)

## References

[1] Canchaya C, Fournous G, Chibani-Chennoufi S, Dillmann ML, Brussow H (2003) Phage as agents of lateral gene transfer. Curr Opin Microbiol 6: 417–424. 12941415 10.1016/s1369-5274(03)00086-9

[2] Chen I, Christie PJ, Dubnau D (2005) The ins and outs of DNA transfer in bacteria. Science 310: 1456–1460. 16322448 10.1126/science.1114021 PMC3919525

[3] Claverys JP, Martin B, Polard P (2009) The genetic transformation machinery: composition, localization, and mechanism. FEMS Microbiol Rev 33: 643–656. 19228200 10.1111/j.1574-6976.2009.00164.x

[4] Spratt BG, Maiden MC (1999) Bacterial population genetics, evolution and epidemiology. Philos Trans R Soc Lond B Biol Sci 354: 701–710. 10365396 10.1098/rstb.1999.0423 PMC1692550

[5] Townsend JP, Nielsen KM, Fisher DS, Hartl DL (2003) Horizontal acquisition of divergent chromosomal DNA in bacteria: effects of mutator phenotypes. Genetics 164: 13–21. 12750317 10.1093/genetics/164.1.13 PMC1462543

[6] Thomas CM, Nielsen KM (2005) Mechanisms of, and barriers to, horizontal gene transfer between bacteria. Nat Rev Microbiol 3: 711–721. 16138099 10.1038/nrmicro1234

[7] Donati C, Hiller NL, Tettelin H, Muzzi A, Croucher NJ, et al. (2010) Structure and dynamics of the pan-genome of Streptococcus pneumoniae and closely related species. Genome Biol 11: R107. 21034474 10.1186/gb-2010-11-10-r107 PMC3218663

[8] Hiller NL, Ahmed A, Powell E, Martin DP, Eutsey R, et al. (2010) Generation of genic diversity among Streptococcus pneumoniae strains via horizontal gene transfer during a chronic polyclonal pediatric infection. PLoS Pathog 6: e1001108. 20862314 10.1371/journal.ppat.1001108 PMC2940740

[9] Croucher NJ, Walker D, Romero P, Lennard N, Paterson GK, et al. (2009) Role of conjugative elements in the evolution of the multidrug-resistant pandemic clone Streptococcus pneumoniaeSpain23F ST81. J Bacteriol 191: 1480–1489. 19114491 10.1128/JB.01343-08 PMC2648205

[10] Brochet M, Rusniok C, Couve E, Dramsi S, Poyart C, et al. (2008) Shaping a bacterial genome by large chromosomal replacements, the evolutionary history of Streptococcus agalactiae. Proc Natl Acad Sci U S A 105: 15961–15966. 18832470 10.1073/pnas.0803654105 PMC2572952

[11] He M, Sebaihia M, Lawley TD, Stabler RA, Dawson LF, et al. (2010) Evolutionary dynamics of Clostridium difficile over short and long time scales. Proc Natl Acad Sci U S A 107: 7527–7532. 20368420 10.1073/pnas.0914322107 PMC2867753

[12] Holden MT, Lindsay JA, Corton C, Quail MA, Cockfield JD, et al. (2010) Genome sequence of a recently emerged, highly transmissible, multi-antibiotic- and antiseptic-resistant variant of methicillin-resistant Staphylococcus aureus, sequence type 239 (TW). J Bacteriol 192: 888–892. 19948800 10.1128/JB.01255-09 PMC2812470

[13] Awadalla P (2003) The evolutionary genomics of pathogen recombination. Nat Rev Genet 4: 50–60. 12509753 10.1038/nrg964

[14] Conway DJ, Roper C, Oduola AM, Arnot DE, Kremsner PG, et al. (1999) High recombination rate in natural populations of Plasmodium falciparum. Proc Natl Acad Sci U S A 96: 4506–4511. 10200292 10.1073/pnas.96.8.4506 PMC16362

[15] Didelot X, Lawson D, Darling A, Falush D (2010) Inference of homologous recombination in bacteria using whole-genome sequences. Genetics 186: 1435–1449. 20923983 10.1534/genetics.110.120121 PMC2998322

[16] Hein J, Schierup MH, Wiuf C (2005) Gene genealogies, variation and evolution : a primer in coalescent theory. Oxford; New York: Oxford University Press.

[17] Hey J, Wakeley J (1997) A coalescent estimator of the population recombination rate. Genetics 145: 833–846. 9055092 10.1093/genetics/145.3.833 PMC1207867

[18] Hudson RR (2001) Two-locus sampling distributions and their application. Genetics 159: 1805–1817. 11779816 10.1093/genetics/159.4.1805 PMC1461925

[19] Betran E, Rozas J, Navarro A, Barbadilla A (1997) The estimation of the number and the length distribution of gene conversion tracts from population DNA sequence data. Genetics 146: 89–99. 9136003 10.1093/genetics/146.1.89 PMC1207963

[20] Smith JM (1992) Analyzing the mosaic structure of genes. J Mol Evol 34: 126–129. 1556748 10.1007/BF00182389

[21] Feil EJ, Holmes EC, Bessen DE, Chan MS, Day NP, et al. (2001) Recombination within natural populations of pathogenic bacteria: short-term empirical estimates and long-term phylogenetic consequences. Proc Natl Acad Sci U S A 98: 182–187. 11136255 10.1073/pnas.98.1.182 PMC14565

[22] Posada D, Crandall KA, Holmes EC (2002) Recombination in evolutionary genomics. Annu Rev Genet 36: 75–97. 12429687 10.1146/annurev.genet.36.040202.111115

[23] Westesson O, Holmes I (2009) Accurate detection of recombinant breakpoints in whole-genome alignments. PLoS Comput Biol 5: e1000318. 19300487 10.1371/journal.pcbi.1000318 PMC2651022

[24] Martin D, Rybicki E (2000) RDP: detection of recombination amongst aligned sequences. Bioinformatics 16: 562–563. 10980155 10.1093/bioinformatics/16.6.562

[25] Martin DP, Lemey P, Lott M, Moulton V, Posada D, et al. (2010) RDP3: a flexible and fast computer program for analyzing recombination. Bioinformatics 26: 2462–2463. 20798170 10.1093/bioinformatics/btq467 PMC2944210

[26] Feil EJ, Li BC, Aanensen DM, Hanage WP, Spratt BG (2004) eBURST: inferring patterns of evolutionary descent among clusters of related bacterial genotypes from multilocus sequence typing data. J Bacteriol 186: 1518–1530. 14973027 10.1128/JB.186.5.1518-1530.2004 PMC344416

[27] Tamura K, Dudley J, Nei M, Kumar S (2007) MEGA4: Molecular Evolutionary Genetics Analysis (MEGA) software version 4.0. Mol Biol Evol 24: 1596–1599. 17488738 10.1093/molbev/msm092

[28] Darling AC, Mau B, Blattner FR, Perna NT (2004) Mauve: multiple alignment of conserved genomic sequence with rearrangements. Genome Res 14: 1394–1403. 15231754 10.1101/gr.2289704 PMC442156

[29] Pearson WR, Lipman DJ (1988) Improved tools for biological sequence comparison. Proc Natl Acad Sci U S A 85: 2444–2448. 3162770 10.1073/pnas.85.8.2444 PMC280013

[30] Oppenheim AV SR (1975) Digital Signal Processing. Englewood Cliffs, NJ: Prentice-All, Inc.

[31] Lingjaerde OC, Baumbusch LO, Liestol K, Glad IK, Borresen-Dale AL (2005) CGH-Explorer: a program for analysis of array-CGH data. Bioinformatics 21: 821–822. 15531610 10.1093/bioinformatics/bti113

[32] Robinson DA, Enright MC (2004) Evolution of Staphylococcus aureus by large chromosomal replacements. J Bacteriol 186: 1060–1064. 14762000 10.1128/JB.186.4.1060-1064.2004 PMC344219

[33] Stefani S, Chung DR, Lindsay JA, Friedrich AW, Kearns AM, et al. (2012) Meticillin-resistant Staphylococcus aureus (MRSA): global epidemiology and harmonisation of typing methods. Int J Antimicrob Agents 39: 273–282. 22230333 10.1016/j.ijantimicag.2011.09.030

[34] Tzeng YL, Datta A, Strole C, Kolli VS, Birck MR, et al. (2002) KpsF is the arabinose-5-phosphate isomerase required for 3-deoxy-D-manno-octulosonic acid biosynthesis and for both lipooligosaccharide assembly and capsular polysaccharide expression in Neisseria meningitidis. J Biol Chem 277: 24103–24113. 11956197 10.1074/jbc.M200931200

[35] Obert C, Sublett J, Kaushal D, Hinojosa E, Barton T, et al. (2006) Identification of a Candidate Streptococcus pneumoniae core genome and regions of diversity correlated with invasive pneumococcal disease. Infect Immun 74: 4766–4777. 16861665 10.1128/IAI.00316-06 PMC1539573

[36] Sanchez-Beato AR, Lopez R, Garcia JL (1998) Molecular characterization of PcpA: a novel choline-binding protein of Streptococcus pneumoniae. FEMS Microbiol Lett 164: 207–214. 9675866 10.1111/j.1574-6968.1998.tb13087.x

[37] Chi F, Nolte O, Bergmann C, Ip M, Hakenbeck R (2007) Crossing the barrier: evolution and spread of a major class of mosaic pbp2x in Streptococcus pneumoniae, S. mitis and S. oralis. Int J Med Microbiol 297: 503–512. 17459765 10.1016/j.ijmm.2007.02.009

[38] Huang YT, Hsueh PR (2008) Antimicrobial drug resistance in Taiwan. Int J Antimicrob Agents 32 Suppl 3: S174–178. 19013350 10.1016/S0924-8579(08)70023-2

[39] Kilian M, Poulsen K, Blomqvist T, Havarstein LS, Bek-Thomsen M, et al. (2008) Evolution of Streptococcus pneumoniae and its close commensal relatives. PLoS ONE 3: e2683. 18628950 10.1371/journal.pone.0002683 PMC2444020

[40] Feng Y, Chen CJ, Su LH, Hu S, Yu J, et al. (2008) Evolution and pathogenesis of Staphylococcus aureus: lessons learned from genotyping and comparative genomics. FEMS Microbiol Rev 32: 23–37. 17983441 10.1111/j.1574-6976.2007.00086.x

[41] Feng J, Lupien A, Gingras H, Wasserscheid J, Dewar K, et al. (2009) Genome sequencing of linezolid-resistant Streptococcus pneumoniae mutants reveals novel mechanisms of resistance. Genome Res 19: 1214–1223. 19351617 10.1101/gr.089342.108 PMC2704432

[42] Boto L (2010) Horizontal gene transfer in evolution: facts and challenges. Proc Biol Sci 277: 819–827. 19864285 10.1098/rspb.2009.1679 PMC2842723

[43] Brueggemann AB, Griffiths DT, Meats E, Peto T, Crook DW, et al. (2003) Clonal relationships between invasive and carriage Streptococcus pneumoniae and serotype- and clone-specific differences in invasive disease potential. J Infect Dis 187: 1424–1432. 12717624 10.1086/374624

[44] Fraser C, Hanage WP, Spratt BG (2007) Recombination and the nature of bacterial speciation. Science 315: 476–480. 17255503 10.1126/science.1127573 PMC2220085

[45] Hanage WP, Fraser C, Tang J, Connor TR, Corander J (2009) Hyper-recombination, diversity, and antibiotic resistance in pneumococcus. Science 324: 1454–1457. 19520963 10.1126/science.1171908

