# Supplementary figures and images for: A Novel Computational Method Identifies Intra- and Inter-Species Recombination Events in Staphylococcus aureus and Streptococcus pneumoniae
Source: PLoS Comput Biol. 2012 Sep 6;8(9):e1002668. doi: 10.1371/journal.pcbi.1002668 (PMC3435249; doi:10.1371/journal.pcbi.1002668)

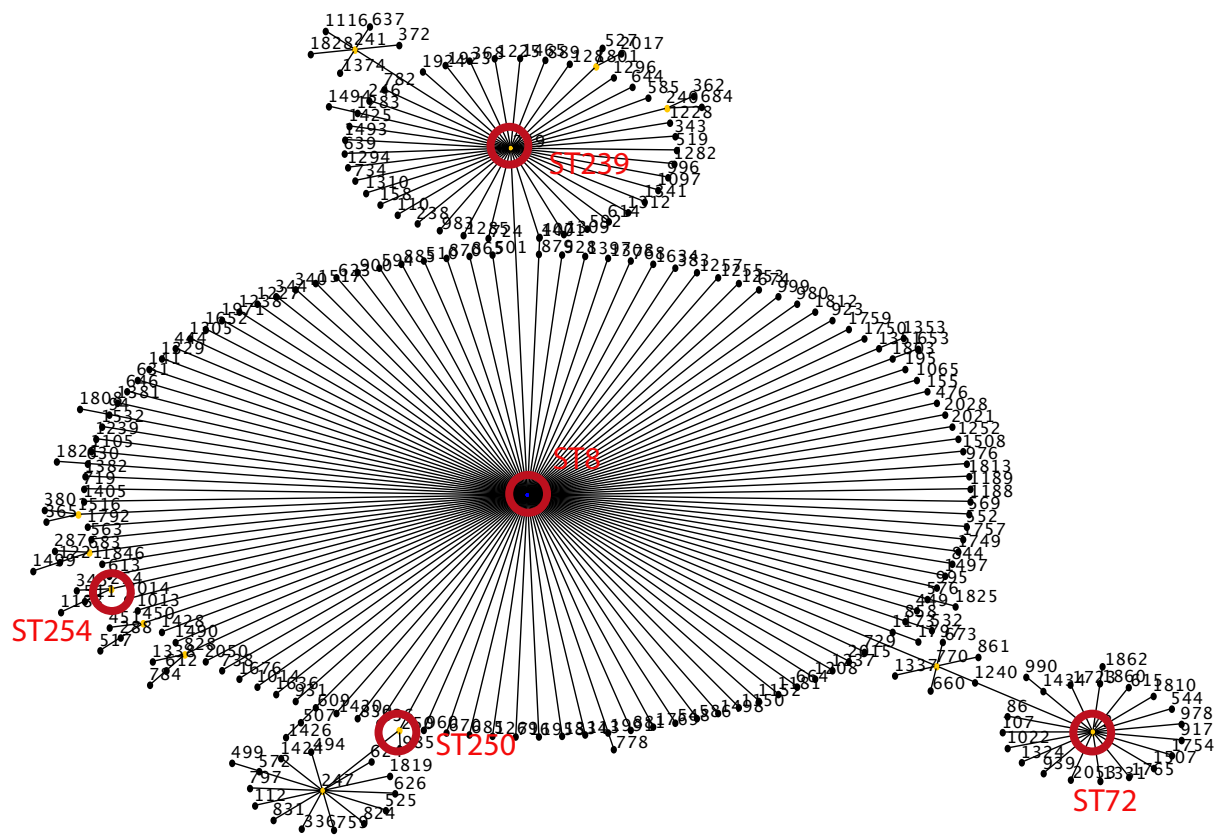

Supplement: Figure S1 — Structure of CC8 of S. aureus . Minimum spanning tree of the CC8 obtained by eBurst on the MLST data. The STs included in our strain collection are indicated. (PDF) [file pcbi.1002668.s001.pdf]

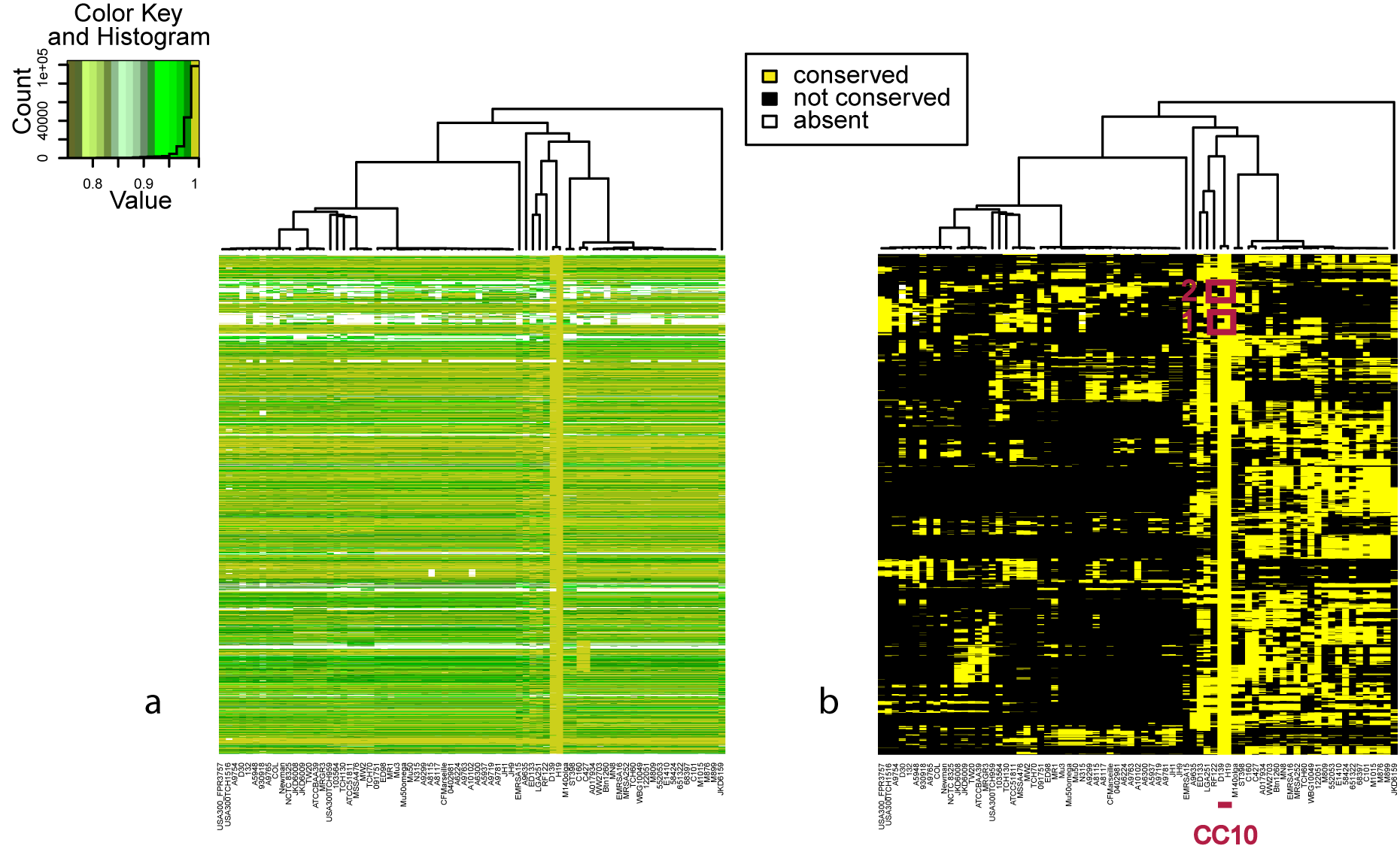

Supplement: Figure S2 — Recombination in S. aureus CC10. a) Comparison of S. aureus H19 (CC10) strain against all the other S. aureus strains. b) Filtered data. Two regions (box 1 and 2) of H19 are not conserved in the other genome of CC10 (D139). These regions include two phages shared with CC10 strains. (TIF) [file pcbi.1002668.s002.tif]

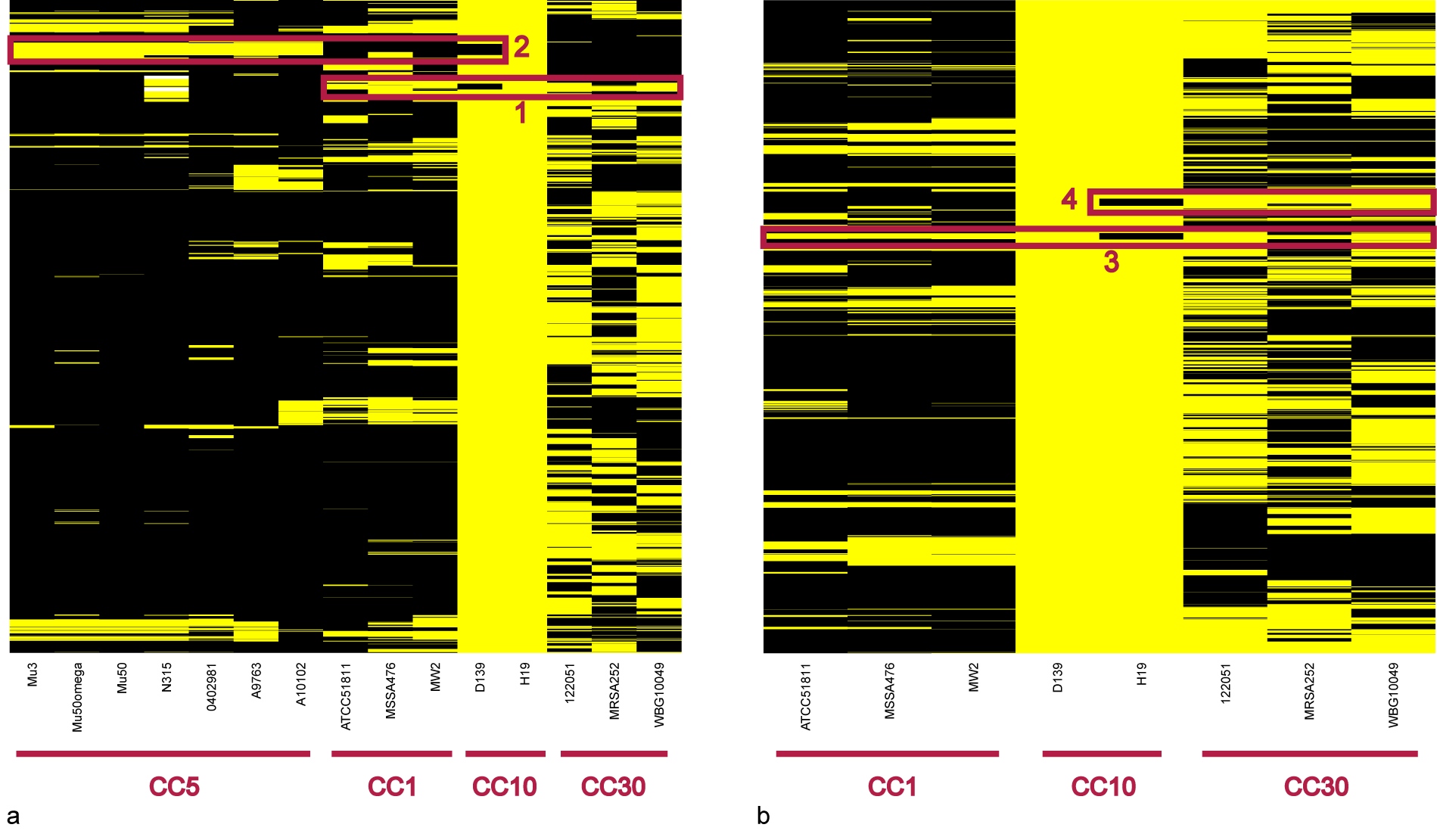

Supplement: Figure S3 — Comparison of H19 with the subset of potential donor genomes of regions 1 and 2 in Fig. S2b. a) Region (1) shows high similarity with genomes that belong to CC30 (122051, MRSA252, WBG10049) or CC1 (ATCC51811, MSSA476, MW2) while region (2) shows high similarity with genomes of CC5 (Mu3, Mu50omega, Mu50, N315, 0402981, A9763, A10102). b) Comparison of D139 with the subset of potential donor genomes. Region (3) was probably acquired from genomes of CC30 (122051,MRSA252, WBG10049) or CC1 (ATCC51811, MSSA476, MW2), while region (4) was probably acquired from genomes of CC30 (122051, MRSA252, WBG10049). (TIF) [file pcbi.1002668.s003.tif]

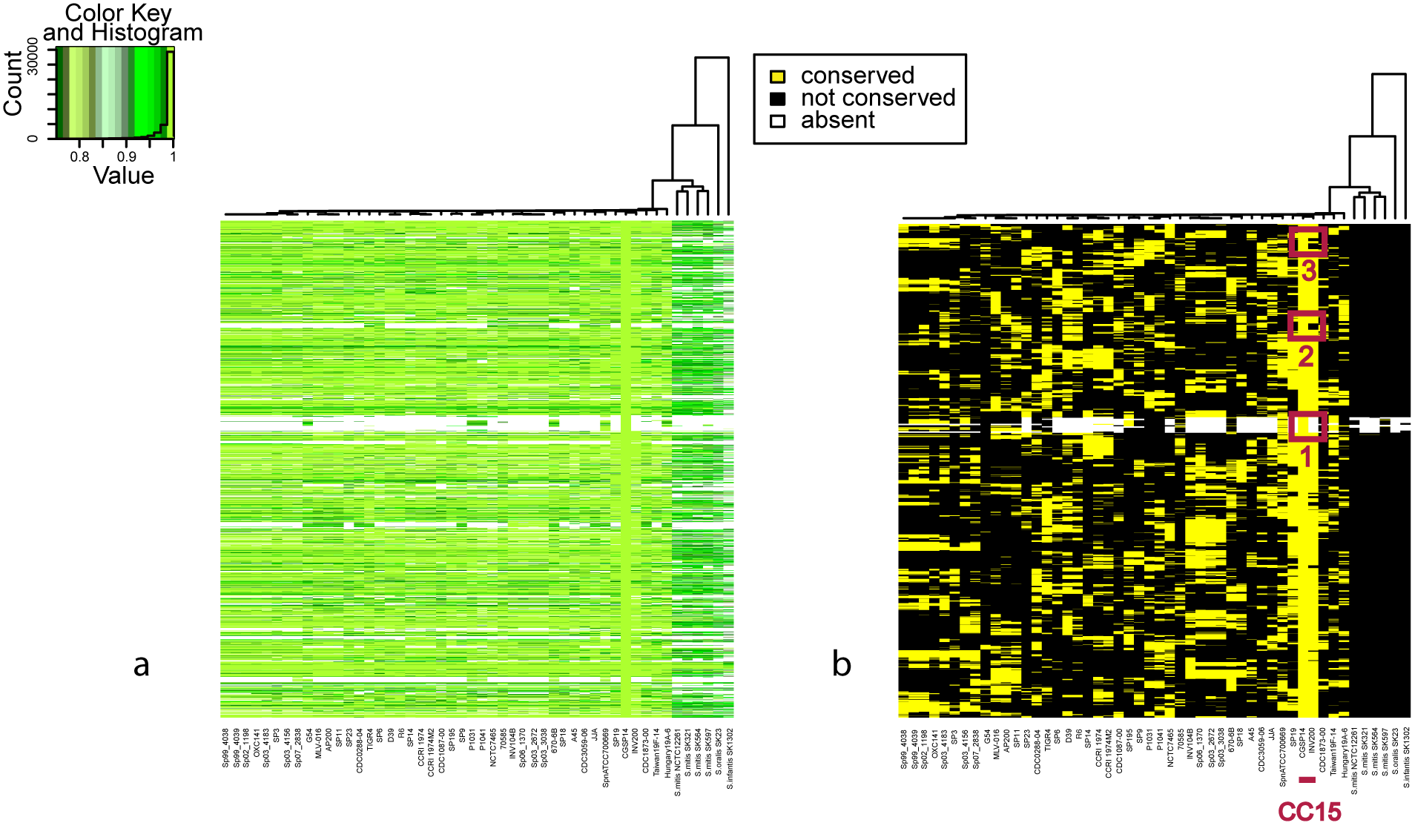

Supplement: Figure S4 — Recombination in S. pneumoniae CC15. a) Comparison of S. pneumoniae CGSP14 (CC15) strain against all the other S. pneumoniae strains. b) Output of the low pass filter. CGSP14 shows mainly three large regions not shared with the other strain INV200 belonging to CC15. Region (1) shows similarity with SpnATCC700669 (CC81, serotype 23F), region (2) with CDC3059-06 (CC199) and TIGR4 (CC205) and region (3) with SpnATCC700669 and most of the genomes of CC180. (TIF) [file pcbi.1002668.s004.tif]
