# Supplementary material for: A Novel Computational Method Identifies Intra- and Inter-Species Recombination Events in Staphylococcus aureus and Streptococcus pneumoniae
Source: PLoS Comput Biol. 2012 Sep 6;8(9):e1002668. doi: 10.1371/journal.pcbi.1002668 (PMC3435249; doi:10.1371/journal.pcbi.1002668)
Supplement: Table S1 — Strain collection of Staphylococcus aureus . (DOC) [file pcbi.1002668.s006.doc]

Table S1. Strain collection of *Staphylococcus aureus*

| **Organism** | **MLST** | **CC** | **Genome(bp)** | **ORFs** | **Center** | **Status** | **Accession number** |
| --- | --- | --- | --- | --- | --- | --- | --- |
| NCTC 8325 | 8 | 8 | 2821361 | 2891 | University of Oklahoma Health Sciences Center | completed | NC_007795 |
| COL | 250 | 8 | 2809422 | 2612 | TIGR | completed | NC_002951 |
| USA300 FPR3757 | 8 | 8 | 2872769 | 2560 | University of California, San Francisco | completed | NC_007793 |
| USA300_TCH1516 | 8 | 8 | 2872915 | 2657 | Baylor college of medicine | completed | NC_010079 |
| Newman | 254 | 8 | 2878897 | 2614 | Juntendo University | completed | NC_009641 |
| A5948 | 8 | 8 | 2846107 | 2682 | Broad Institute | draft | NZ_ACKD00000000 |
| 132 | 8 | 8 | 2825358 | 2693 | Instituto de Agrobiotecnologia Universidad Publica de Navarra | draft | NZ_ACOT00000000 |
| TCH130 | 72 | 8 | 2765336 | 2856 | Baylor College of Medicine | draft | NZ_ACHD00000000 |
| JKD6008 | 239 | 8 | 2924344 | 2680 | Monash University | completed | CP002120 |
| JKD6009 | 239 | 8 | 2819448 | 2684 | Monash University | draft | NZ_ABSA00000000 |
| TW20 | 239 | 8 | 3043210 | 2809 | Sanger Institute | completed | FN433596 |
| MRGR3* | 239 | 8 |  |  |  | draft |  |
| ATCC BAA-39 | 239 | 8 | 2865318 | 2836 | Baylor College of Medicine | draft | NZ_AEEK00000000 |
| 930918-3 | 8 | 8 | 2923460 | 2142 | University of Central Florida | draft | NZ_ABFA00000000 |
| D30 | 8 | 8 | 2775621 | 2281 | University of Central Florida | draft | NZ_ABFB00000000 |
| A9765 | 8 | 8 | 2887954 | 2877 | Broad Institute | draft | NZ_ACSN00000000 |
| A9754 | 8 | 8 | 2930317 | 2866 | Broad Institute | draft | NZ_ADJI00000000 |
| JH1 | 105 | 5 | 2906507 | 2747 | US DOE Joint Genome Institute | completed | NC_009632 |
| JH9 | 105 | 5 | 2906700 | 2697 | US DOE Joint Genome Institute | completed | NC_009487 |
| Mu3 | 5 | 5 | 2880168 | 2690 | Juntendo University | completed | NC_009782 |
| Mu50 | 5 | 5 | 2878529 | 2696 | Juntendo University | completed | NC_002758 |
| N315 | 5 | 5 | 2814816 | 2583 | Juntendo University/NITE | completed | NC_002745 |
| ED98 | 5 | 5 | 2824404 | 2661 | University of Edinburgh | completed | NC_013450 |
| A5937 | 5 | 5 | 2827272 | 2706 | Broad Institute | draft | NZ_ACKC00000000 |
| A6224 | 5 | 5 | 2870228 | 2755 | Broad Institute | draft | NZ_ACKE00000000 |
| A6300 | 5 | 5 | 2816649 | 2674 | Broad Institute | draft | NZ_ACKF00000000 |
| A8115 | 5 | 5 | 2746960 | 2539 | Broad Institute | draft | NZ_ACKG00000000 |
| A9299 | 5 | 5 | 2782181 | 2625 | Broad Institute | draft | NZ_ACKH00000000 |
| A9719 | 5 | 5 | 2856961 | 2760 | Broad Institute | draft | NZ_ACKJ00000000 |
| A9763 | 5 | 5 | 2769858 | 2605 | Broad Institute | draft | NZ_ACKK00000000 |
| A9781 | 5 | 5 | 2822169 | 2660 | Broad Institute | draft | NZ_ACKL00000000 |
| Mu50-omega | 5 | 5 | 2878428 | 2680 | Juntendo University | draft | NZ_BABM00000000 |
| CF-Marseille | 5 | 5 | 2829971 | 2708 | Geneva University Hospitals | draft | NZ_CABA00000000 |
| 091751* | 5 | 5 |  |  |  | draft |  |
| A10102 | 5 | 5 | 2820632 | 2742 | Broad Institute | draft | NZ_ACSO00000000 |
| A8117 | 5 | 5 | 2715851 | 2626 | Broad Institute | draft | NZ_ACYO00000000 |
| 04-02981 | 225 | 5 | 2821452 | 2652 | Robert Koch Institute | completed | CP001844 |
| MR1 | 5 | 5 | 2808969 | 2775 | University of Edinburgh | draft | NZ_ACZQ00000000 |
| MRSA252 | 36 | 30 | 2902619 | 2650 | Sanger Institute | completed | NC_002952 |
| 55/2053 | 30 | 30 | 2762249 | 2648 | Broad Institute | draft | NZ_ACJR00000000 |
| 65-1322 | 30 | 30 | 2777290 | 2629 | Broad Institute | draft | NZ_ACJS00000000 |
| 68-397 | 30 | 30 | 2767646 | 2621 | Broad Institute | draft | NZ_ACJT00000000 |
| E1410 | 30 | 30 | 2810484 | 2647 | Broad Institute | draft | NZ_ACJU00000000 |
| M876 | 30 | 30 | 2762708 | 2606 | Broad Institute | draft | NZ_ACJV00000000 |
| MN8 | 30 | 30 | 2882664 | 2833 | Baylor College of Medicine | draft | NZ_ACJA00000000 |
| TCH60 | 30 | 30 | 2802675 | 2703 | Baylor College of Medicine | completed | CP002110 |
| A017934/97 | 30 | 30 | 2801838 | 2627 | Broad Institute | draft | NZ_ACYP00000000 |
| Btn1260 | 30 | 30 | 2780206 | 2591 | Broad Institute | draft | NZ_ACUU00000000 |
| C101 | 30 | 30 | 2781692 | 2687 | Broad Institute | draft | NZ_ACSP00000000 |
| C160 | 34 | 30 | 2822646 | 2750 | Broad Institute | draft | NZ_ACUV00000000 |
| M899 | 30 | 30 | 2768593 | 2682 | Broad Institute | draft | NZ_ACSU00000000 |
| WBG10049 | 30 | 30 | 2777175 | 2581 | Broad Institute | draft | NZ_ACSV00000000 |
| WW2703/97 | 30 | 30 | 2743583 | 2621 | Broad Institute | draft | NZ_ACSW00000000 |
| 122051* | 30 | 30 |  |  |  | draft |  |
| 58-424 | 30 | 30 | 2798503 | 2624 | Broad Institute | draft | NZ_ACUT00000000 |
| M1015 | 30 | 30 | 2764225 | 2686 | Broad Institute | draft | NZ_ACST00000000 |
| EMRSA16 | 36 | 30 | 2879203 | 2733 | Broad Institute | draft | NZ_ADAT00000000 |
| TCH70 | 1 | 1 | 2812425 | 2730 | Baylor College of Medicine | draft | NZ_ACHH00000000 |
| MSSA476 | 1 | 1 | 2799802 | 2571 | Sanger Institute | completed | NC_002953 |
| MW2 | 1 | 1 | 2820462 | 2624 | <NITE> | completed | NC_003923 |
| ATCC 51811 | 1 | 1 | 2765363 | 2693 | Baylor College of Medicine | draft | NZ_ADVP00000000 |
| D139 | 145 | 10 | 2795023 | 2721 | Broad Institute | draft | NZ_ACSR00000000 |
| H19 | 10 | 10 | 2783181 | 2628 | Broad Institute | draft | NZ_ACSS00000000 |
| A9635 | 278 | 45 | 2731263 | 2592 | Broad Institute | draft | NZ_ACKI00000000 |
| USA300 TCH959 | 1159 | 7 | 2810505 | 2906 | Baylor College of Medicine | draft | NZ_AASB00000000 |
| C427 | 42 | singl | 2861691 | 2781 | Broad Institute | draft | NZ_ACSQ00000000 |
| EMRSA15 | 22 | 22 | 2832299 | 2602 | Sanger Institute | completed | ftp.sanger.ac.uk/pub/pathogens/sa/ |
| 103564* | 80 | 80 |  |  |  | draft |  |
| LGA251 ^1^ | 425 | 425 | 2750834 | 2433 | Sanger Institute | complete | FR821779 |
| M140olga ^2^* | 398 | 398 |  |  |  | draft |  |
| ST398 | 398 | 398 | 2872582 | 2746 | University Medical Center Utrecht | completed | AM990992 |
| RF122  ^3^ | 151 | 705 | 2742531 | 2509 | University of Minnesota | completed | NC_007622 |
| ED133 ^4^ | 1452 | 133 | 2832478 | 2653 | University of Edinburgh | completed | CP001996 |
| JKD6159 | 93 | 93 | 2811435 | 2577 | University of Melbourne | completed | CP002114 |
| M809 | 431 | 30 | 2770328 | 2673 | Broad Institute | draft | NZ_ACUS00000000 |

1,3 Strain from cattle

2 Strain from swine

4 Strain from sheep

* Newly sequenced strains
