# Supplementary material for: A Novel Computational Method Identifies Intra- and Inter-Species Recombination Events in Staphylococcus aureus and Streptococcus pneumoniae
Source: PLoS Comput Biol. 2012 Sep 6;8(9):e1002668. doi: 10.1371/journal.pcbi.1002668 (PMC3435249; doi:10.1371/journal.pcbi.1002668)
Supplement: Table S2 — Newly sequenced strains of Staphylococcus aureus . (DOC) [file pcbi.1002668.s007.doc]

Table S2. Newly sequenced strains of Staph*ylococcus aureus*

| **Strain** | **Location of isolation** | **Year** | **Carriage/ Disease** | **N° of contigs** | **Size (Mb)** | **G + C content (%)** | **ST** | **N° of genes** | **Technology** |
| --- | --- | --- | --- | --- | --- | --- | --- | --- | --- |
| MRGR3 | Geneva | 1979 | Sepsis on CVC | 173 | 3.02 | 32.63 | 239 | 3066 | Illumina Solexa |
| 091751 | Geneva | 2000 | Nasal colonization (neonate) | 210 | 2.84 | 32.66 | 5-PVL | 2911 | Illumina Solexa |
| 122051 | Geneva | 2005 | Pus | 226 | 2.77 | 32.66 | 30 PVL | 2800 | Illumina Solexa |
| 103564 | Geneva | 2002 | Furonculosis | 222 | 2.77 | 32.67 | 80 PVL | 2774 | Illumina Solexa |
| 119857 | Geneva | 2008 | Nasal colonization | 130 | 2.78 | 32.79 | 398 | 2787 | Illumina Solexa |
