# Supplementary material for: A Novel Computational Method Identifies Intra- and Inter-Species Recombination Events in Staphylococcus aureus and Streptococcus pneumoniae
Source: PLoS Comput Biol. 2012 Sep 6;8(9):e1002668. doi: 10.1371/journal.pcbi.1002668 (PMC3435249; doi:10.1371/journal.pcbi.1002668)
Supplement: Table S3 — Strain collection of Streptococcus pneumoniae . (DOCX) [file pcbi.1002668.s008.docx]

| **Species** | **Strain** | **Serotype** | **MLST** | **CC** | **Genome (bp)** | **#ORFs** | **Location of isolation** | **Status** | **Accession Number** |
| --- | --- | --- | --- | --- | --- | --- | --- | --- | --- |
| S.pneumoniae | SP3 | 3 | 180 | CC180 | 2033581 | 2177 | Pittsburgh, US | draft | GenBank:AAZZ00000000 |
|  | SP6 | 6 | 460 | CC460 | 2162916 | 2325 | Pittsburgh, US | draft | GenBank:ABAA00000000 |
|  | SP9 | 9 | 1269 | CC156 | 2117908 | 2241 | Pittsburgh, US | draft | GenBank:ABAB00000000 |
|  | SP11 | 11 | 62 | CC62 | 2060705 | 2127 | Pittsburgh, US | draft | GenBank:ABAC00000000 |
|  | SP14 | 14 | 124 | CC124 | 2148093 | 2624 | Pittsburgh, US | draft | GenBank:ABAD00000000 |
|  | SP18 | 6 | new | CC90 | 2105593 | 2211 | Pittsburgh, US | draft | GenBank:ABAE00000000 |
|  | SP19 | 19 | 485 | CC395 | 2136434 | 2301 | Pittsburgh, US | draft | GenBank:ABAF00000000 |
|  | SP23 | 23 | 37 | CC439 | 2103479 | 2200 | Pittsburgh, US | draft | GenBank:ABAG00000000 |
|  | INV104B | 1 | 227 | CC306 | 2142122 | 1941 | Oxford, UK | complete | GenBank:FQ312030 |
|  | OXC141 | 3 | 180 | CC180 | 2036967 | 1973 | Oxford, UK | complete | GenBank:FQ312027 |
|  | INV200 | 14 | 9 | CC15 | 2093318 | 2045 | Oxford, UK | complete | GenBank:FQ312029 |
|  | SpnATCC700669 | 23F | 81 | CC81 | 2221315 | 2132 | Spain | complete | GenBank:FM211187 |
|  | Sp03_4156 | 3 | 180 | CC180 | 2058353 | 1954 | The Netherlands | draft | GenBank:FQ312045 |
|  | Sp03_4183 | 3 | 180 | CC180 | 1993183 | 1933 | The Netherlands | draft | GenBank:FQ312043 |
|  | Sp07_2838 | 3 | 180 | CC180 | 1990038 | 1901 | Bolivia | draft | GenBank:CACI01000000 |
|  | Sp99_4038 | 3 | 180 | CC180 | 2010908 | 1952 | Glasgow Reference Lab | draft | GenBank:FQ312041 |
|  | Sp99_4039 | 3 | 180 | CC180 | 2010104 | 1954 | Glasgow Reference Lab | draft | GenBank:FQ312044 |
|  | Sp02_1198 | 3 | 180 | CC180 | 1989367 | 1938 | Glasgow Reference Lab | draft | GenBank:CACH01000000 |
|  | A45 | 3 | New |  | 2041833 | 1932 | Newmarket | draft | GenBank:CACG01000000 |
|  | P1041 | 1 | 217 | CC217 | 2166490 | 1905 | Ghana | draft | GenBank:CACE01000000 |
|  | Sp03_2672 | 1 | 306 | CC306 | 2144331 | 1904 | Glasgow Reference Lab | draft | GenBank:FQ312039 |
|  | Sp03_3038 | 1 | 306 | CC306 | 2164519 | 1936 | Glasgow Reference Lab | draft | GenBank:FQ312042 |
|  | Sp06_1370 | 1 | 306 | CC306 | 2012346 | 1874 | Glasgow Reference Lab | draft | GenBank:CACJ01000000 |
|  | NCTC7465 | 1 | 615 | CC2296 | 2100988 | 1845 | Type strain, Rockefeller USA, 1948 | draft | GenBank:CACF01000000 |
|  | P1031 | 1 | 303 | CC217 | 2111882 | 2073 | Ghana | complete | GenBank:CP000920 |
|  | D39 | 2 | 595 | CC128 | 2046115 | 1914 | US | complete | GenBank:CP000410 |
|  | TIGR4 | 4 | 205 | CC205 | 2160842 | 2125 | Norway | complete | GenBank:AE005672 |
|  | 70585 | 5 | 289 | CC289 | 2184682 | 2202 | Bangladesh | complete | GenBank:CP000918 |
|  | JJA | 14 | 66 | CC66 | 2120234 | 2123 | Brazil | complete | GenBank:CP000919 |
|  | MLV-016 | 11A | 62 | CC62 | 2247118 | 2159 | USA, Europe | draft | GenBank:ABGH00000000 |
|  | CDC0288-04 | 12F | 220 | CC218 | 2051140 | 2105 | USA, UK | draft | GenBank:ABGF00000000 |
|  | CDC3059-06 | 19A | 199 | CC199 | 2293277 | 2379 | Iceland, UK, USA, others | draft | GenBank:ABGG00000000 |
|  | Hungary19A-6 | 19A | 268 | CC176 | 2245615 | 2155 | Hungary | complete | GenBank:CP000936 |
|  | Taiwan19F-14 | 19F | 236 | CC271 | 2112148 | 2044 | Taiwan | complete | GenBank:CP000921 |
|  | CDC1873-00 | 6A | 376 | CC2090 | 2265195 | 2402 | USA | draft | GenBank:ABFS00000000 |
|  | 670-6B | 6B | 90 | CC90 | 2240045 | 2384 | Spain | complete | GenBank: CP002176 |
|  | CDC1087-00 | 7F | 191 | CC191 | 2190853 | 2232 | Bra, Den, Fin, Neth, Nor, UK, Uru, USA | draft | GenBank:ABFT00000000 |
|  | SP195 | 9V | 156 | CC156 | 2198294 | 2287 | Worldwide | draft | GenBank:ABGE00000000 |
|  | G54 | 19F | 63 | CC63 | 2078953 | 2115 | Italy | complete | GenBank:CP001015 |
|  | R6 | 2 | 595 | CC128 | 2038615 | 2043 | Laboratory | complete | GenBank:AE007317 |
|  | CGSP14 | 14 | 15 | CC15 | 2209198 | 2206 | China, Beijing Institute of Genomics | complete | GenBank:CP001033 |
|  | CCRI 1974 | 14 | 124 | CC124 | 2005075 | 2074 | McGill University, Canada | draft | GenBank:ABZC00000000 |
|  | CCRI 1974M2 | 14 | 124 | CC124 | 2003231 | 2069 | McGill University, Canada | draft | GenBank:ABZT00000000 |
|  | AP200 | 11A | 62 | CC62 | 2084139 | 2149 | Italy | draft | GenBank:CP002121 |
| S. mitis | S. mitis NCTC12261 | / | / | / | 1831909 | 1742 | Denmark | draft | GenBank:AEDX00000000** |
|  | S. mitis SK321 | / | / | / | 1884262 | 1855 | Denmark | draft | GenBank:AEDT00000000** |
|  | S. mitis SK564 | / | / | / | 2036999 | 1997 | Denmark | draft | GenBank:AEDU00000000** |
|  | S. mitis SK597 | / | / | / | 2043724 | 2005 | Denmark | draft | GenBank:AEDV00000000** |
| S. oralis | S. oralis SK23 (NCTC11427/ ATCC35037, type strain) | / | / | / | 1895679 | 1876 | Denmark | draft | GenBank:AEDW00000000** |
| S. infantis | S. infantis SK1302 | / | / | / | 1802590 | 2385 | Denmark | draft | GenBank:AEDY00000000** |
