# Supplementary material for: A Novel Computational Method Identifies Intra- and Inter-Species Recombination Events in Staphylococcus aureus and Streptococcus pneumoniae
Source: PLoS Comput Biol. 2012 Sep 6;8(9):e1002668. doi: 10.1371/journal.pcbi.1002668 (PMC3435249; doi:10.1371/journal.pcbi.1002668)
Supplement: Text S1 — Help file for the Reco package. (DOC) [file pcbi.1002668.s012.doc]

***Reco***

The package Reco contains two main functions, namely identify.reco, that performs the filtering procedure and identifies the borders of the putative recombination events, and draw_heatmap, that produces the graphical output. The two functions are independent, but are typically applied sequentially. The input dataset is composed by a matrix containing the results of a homology search of one test genome against a set of reference genomes, in columnar format For each gene in the test genome, the input must contain one line for each reference genome reporting the percentage of identity of the best hit. If no hit was found for a given reference genome, the value “0” for the percentage of identity should be used.

**Recombination detection (identify.reco)**

Function for the identification of recombination events affecting a group of adjacent genes in a genomic sequence.

The function identifies variations on the percentage of conservation of a set of neighbouring genes between the test genome and the other reference genomes in the dataset.

The function provides genomes that are the donor for the recombination event and a list of genes corresponding with recombination breakpoints.

**INPUT**

matrix of pair comparison (load ‘data(m)’ for example)

**Input file format**

matrix of all pair comparisons as determined by the Fasta alignment. Each pair comparison must have the same number of instances (the best hit for each gene and zero for genes that haven't found homologous) in the same order as in the test genome.

The matrix reports in the first column the genes of the test genome as character, in second column the name of reference genomes as character, in the third column the name of test genome as character, in the fourth column the percentage of identity as numeric. If no hit is found , the value “0” must be used.

**Load matrix**

Syntax

m<-as.matrix(read.table("where_is_matrix/matrix.txt",header=F))

**Run identify.reco**

Syntax

out<-identify.reco(m,round=5, l=20,l_break=20,q=0.75,method="rect")

Command line arguments

‘m’ matrix of all pair comparisons (see above for file format)

‘round’ is the number of decimal places of the weighted mean.

‘l’ is the length of the sliding window in term of genes number. The size of l must be tuned according to the size of the recombination events that we want to identify. For instance the size of l must be set to a value no larger than the region to detect and therefore the user should carefully evaluate different values of this parameter.

‘l_break’ is the length of the sliding window for detecting the breakpoint position. It is usually set of the same size as l.

‘q’ is the statistical cut-off for the identification of the putative major and minor parents in the sliding window.

The optimal value of q is indirectly influenced by the number and genetic relatedness of the genomes included in the analysis. A lower value of q should be set to correct for the sampling bias when the dataset includes closely related sequences.

The ‘method’ represents the weighted function to be used. This should be one of "rect", "norm", "sinc".

**OUTPUT**

out[[1]] is the filtered matrix to be represented by the function ‘draw_heatmap’

out[[2]] is the p-value matrix

out[[3]] is an object of class list with a list of breakpoint positions

out[[4]] matrix of percentage of identity before filtering to be represented by ‘draw_heatmap’

**Write p-value matrix**

Syntax

write.table(out[[2]], file="where_to_save/pvalue_mat.txt", sep="\t")

**Write list of breakpoint positions**

Syntax

sink(file="where_to_save/min_list.txt")

out[[3]]

sink()

**Graphical visualization (draw_heatmap)**

The function ‘draw.heatmap’ allows the visualization of the raw and filtered matrix to identify the putative recombination events in one test genome against the other genomes.

**INPUT**

**dd** the whole-genome phylogenetic tree for columns reordering

**out[[1]]** the filtered matrix

or

**out[[4]]** the matrix of percentage of identity before filtering

**Load tree**

Syntax

library(ape)

tree<-read.tree(tree)

tree1<-as.hclust(tree)

dd<-as.dendrogram(tree1)

Syntax of draw_heatmap

draw_heatmap(out[[4]],Rowv=NA,Colv=dd,dendrogram="column", scale="none",trace="none", col=greens[1:18], mar=c(9,8),cexCol=0.8)

draw.heatmap(out[[1]], Rowv=NA, Colv=dd, dendrogram=”column”, scale="none", col=c(1,7), mar=c(9,8),cexCol=0.8)

Command line arguments

‘out[[4]]’ ‘out[[1]]’ matrix before and after filtering.

‘Rowv’ determines if and how the row dendrogram should be reordered. By default, it is TRUE, which implies dendrogram is computed and reordered based on row means. If NULL or FALSE, then no dendrogram is computed and no reordering is done. If a dendrogram, then it is used "as-is", ie without any reordering. If a vector of integers, then dendrogram is computed and reordered based on the order of the vector.

‘Colv’ determines if and how the column dendrogram should be reordered. It has the options as the Rowv argument above.

‘dendrogram’ character string indicating whether to draw 'none', 'row', 'column' or 'both' dendrograms. Defaults to 'both'. However, if Rowv (or Colv) is FALSE or NULL and dendrogram is 'both', then a warning is issued and Rowv (or Colv) arguments are honoured.

‘scale’ character indicating if the values should be centered and scaled in either the row direction or the column direction, or none. The default is "row" if symm false, and "none" otherwise.

‘col’ colors used for the image. Defaults to heat colors (heat.colors).

‘mar’ numeric vector of length 2 containing the margins for column and row names, respectively.

‘cexCol’ positive numbers, used as cex.axis for the row or column axis labeling. The defaults currently only use number of rows or columns, respectively.

**OUTPUT**

Heatmap representation of the percentage of conservation of the genes of one test genome against the others before filtering (out[[4]]) or conserved and not conserved genes after filtering (out[[1]]).

Each row represents the percentage of identity (or conservation and not conservation) of one gene of test genome in all other strains. Rows are ordered according to the gene order in the test genome.

Each column represents one reference genome. The columns are reordered according to a whole-genome phylogenetic tree.

**Notes**

For other ‘Command line arguments’ and minor functions/details see the manual in the R package.

**Disclaimer**

Novartis Vaccines and Diagnostics and its employees make no warranty or condition of any kind, express, implied or otherwise, with respect to the accuracy, completeness or performance of the software.
